# Genetic diversity and camphor profiling of *Curcuma caesia* roxb.: identification of elite genotypes and in silico prediction of gastroprotective mechanisms

**DOI:** 10.3389/fphar.2026.1730520

**Published:** 2026-04-15

**Authors:** V. Isha, K. Venkatesan, N. Senthil, V. Rajasree, R. Renuka, K. Chandrakumar, S. Vellaikumar, P. Karthikeyan

**Affiliations:** 1 Department of Spices and Plantation Crops, Horticulture College and Research Station (HC&RI), Tamil Nadu Agricultural University (TNAU), Coimbatore, Tamil Nadu, India; 2 Centre for Plant Molecular Biology and Biotechnology (CPMB&B), Tamil Nadu Agricultural University (TNAU), Coimbatore, Tamil Nadu, India; 3 Department of Plant Biotechnology, Tamil Nadu Agricultural University (TNAU), Coimbatore, Tamil Nadu, India; 4 Department of Plant Biochemistry, Centre for Plant Molecular Biology and Biotechnology, Tamil Nadu Agricultural University (TNAU), Coimbatore, Tamil Nadu, India; 5 Department of Plant Biotechnology, Centre for Plant Molecular Biology and Biotechnology (CPMB&B), Tamil Nadu Agricultural University (TNAU), Coimbatore, Tamil Nadu, India; 6 Spinco Tech Pvt Ltd., Chennai, Tamil Nadu, India

**Keywords:** anti-ulcer, camphor, *Curcuma caesia*, genetic diversity, molecular dynamics, phytochemicals

## Abstract

*Curcuma caesia* Roxb (Black Turmeric) is an underexploited medicinal species with recognized ethnobotanical use in gastrointestinal disorders. This study integrates morphological characterization, validated camphor quantification, and computational anti-ulcer evaluation across 21 genotypes collected from diverse Indian agro-climatic zones. Genotypes were cultivated at Coimbatore and Bhavanisagar and characterized using DUS guidelines. Significant morphological variation was observed in plant height (27.81–107.08 cm), rhizome traits, and essential oil yield (0.20%–0.62%), indicating high genetic diversity. GC-MS profiling identified 30 bioactive metabolites, with camphor emerging as the predominant constituent. Quantification using D-camphor standard revealed substantial genotypic variation in camphor content, ranging from 5.19% (GKK-6) to 33.84% (GTE-18), with high concentrations also recorded in GMR-2 (30.35%), GMN-10 (26.41%), and GCA-5 (24.44%). Molecular docking studies against ulcer-associated targets predicted camphor’s superior binding affinity for MMP9 (−7.8 kcal/mol) compared to ranitidine (−6.3 kcal/mol), with stable hydrogen bonding interactions involving TYR A:111 and TYR A:458. Molecular dynamics simulations over 100 ns confirmed stable camphor-MMP9 interactions with minimal RMSD deviation (0.20–0.25 nm) and persistent hydrogen bonding, providing computational support for investigating its potential gastroprotective role in mucosal healing. While *in silico* predictions suggest plausible mechanisms aligning with traditional ethnomedicinal use, these computational findings require experimental validation through *in vitro* enzyme inhibition assays and *in vivo* gastric ulcer models. This study provides a phytochemical and computational foundation for hypothesis-driven investigation of camphor’s potential gastroprotective properties, identifying elite *C. caesia* genotypes (GTE-18, GMR-2, GMN-10) for breeding programs targeting enhanced camphor content and subsequent biological validation.

## 1 Introduction

Black turmeric (*Curcuma caesia* Roxb.) (2n = 42) is an underexploited perennial rhizomatous botanical drug belonging to the family zingiberaceae and commonly known as “Kali Haldi”. It holds a distinct place among botanical drug due to its ethnobotanical significance and rich phytochemical profile. Morphologically it is distinguishable from common turmeric (*Curcuma longa*) by its bluish-black rhizome and deep violet-red leaf midrib. These rhizomes emit a characteristic camphoraceous aroma and are known to contain a high concentration of volatile oils and secondary metabolites (Mahanta et al., 2023). It was first utilized by tribal populations in Assam, Arunachal Pradesh, Manipur, and possibly extending into Myanmar and Thailand regions, tribal communities recognized the medicinal and ritualistic value of *Curcuma caesia* long before formal botanical documentation (Chattopadhyay et al., 2018).

Over subsequent centuries, the cultivation of black turmeric expanded geographically, adapting to varied environmental conditions across Indian states including Odisha, West Bengal, Chhattisgarh, Madhya Pradesh, and southern regions such as Kerala and Tamil Nadu, where it found suitable niches in well-drained soils under partial shade with adequate humidity (Rajkumari and Sanatombi, 2017), While its primary cultivation, overharvesting and rising commercial demand have led to its inclusion in the list of endangered species by the Indian Forest Department (Venugopal et al., 2017). This geographic dispersion resulted in notable regional variations in morphological and phytochemical characteristics, with distinct differences observed in rhizome pigmentation intensity, aromatic profiles, and essential oil composition across populations (Benya et al., 2023). These variations reflect underlying genetic and chemotypic diversity shaped by local selection pressures, environmental factors, and reproductive isolation among geographically separated populations.

*Curcuma caesia* rhizomes are widely used in traditional medicine systems like Ayurveda, Siddha, and tribal practices across India and Southeast Asia (Council of Scientific and Industrial Research, 1972). Rhizomes are rich in volatile oils and secondary metabolites like terpenoids, flavonoids, and curcuminoids, contributing to their gastroprotective, anti-inflammatory, antioxidant, and antimicrobial activities (Mahanta et al., 2023). These properties are particularly effective against gastric ulcers caused by oxidative stress, *Helicobacter pylori*, or NSAID exposure (Tarnawski et al., 2014). Their nutrient-rich composition, including essential minerals and macronutrients, further supports their therapeutic value (Bhardwaj et al., 2023). Traditionally, it has been used to treat asthma, bronchitis, epilepsy, wounds, fevers, gastrointestinal disorders, skin infections, infertility, and inflammatory diseases (Sahu and Saxena, 2014). Their antibacterial properties also support wound healing and infection prevention (Liu et al., 2013), and they are consumed post-surgery to aid clotting and recovery, particularly by women after childbirth (Paw et al., 2020). In tribal medicine, rhizome paste is applied externally for rheumatic pain, sprains, bruises, psoriasis, and skin diseases (Angel et al., 2012). In Manipur, it is used as a pain reliever from bruises and contusions (Sarangthem and Haokip, 2010). The Adi tribe in Arunachal Pradesh uses a decoction to treat diarrhoea (Kagyung et al., 2010), while the Khamti tribe of Lohit district applies rhizome paste for snake and scorpion bites (Tag et al., 2007). The aromatic, camphor-rich tubers are used in cosmetics and by pharmaceutical industries for conditions like Alzheimer’s and inflammatory bowel diseases (Benya et al., 2023), Fresh rhizome is crunched and used as paste for sprains and bruises as well as migraine reliever (Ibrahim et al., 2023). Additionally, dried leaves are used as fuel and to stimulate rice germination, reflecting the plants broad utility (Lalitha et al., 1995).

Genetic diversity within *C. caesia* plays a crucial role in shaping its morphological and molecular variations among genotypes that affect essential oil content and bioactive metabolites. This genetic variability necessitates thorough phytochemical profiling to identify superior genotypes with enhanced therapeutic efficacy, for which Gas Chromatography-Mass Spectrometry (GC-MS) serves as a powerful analytical tool, enabling precise identification and quantification of complex volatile and non-volatile metabolites (Chitra et al., 2020). By coupling GC-MS with computational approaches like molecular docking and molecular dynamics simulations, researchers can gain detailed insights into how key phytochemicals, particularly camphor, the principal bioactive constituent of *C. caesia* rhizomes, interact at the molecular level with protein targets related to gastric ulceration and other disorders, thereby supporting natural therapeutic discovery (Bohra et al., 2021). This integrated methodology of combining genetic diversity assessment with GC-MS profiling and *in silico* molecular interaction studies thus provides a comprehensive framework to validate and advance the pharmacological potential of black turmeric.

Despite *C. caesia* medicinal significance in gastrointestinal therapeutics, its pharmaceutical development faces four critical knowledge deficits. First, systematic phytochemical characterization across geographically diverse germplasm is absent, precluding identification of elite chemotypes for breeding programs, with existing studies reporting inconsistent camphor estimates (4.3%–28.3%) due to semi-quantitative methods lacking authenticated standards (Mukunthan et al., 2014; Pandey et al., 2013). Second, the relationship between camphor content and gastroprotective efficacy remains unexamined through integrated computational-experimental frameworks. Third, molecular mechanisms underlying gastroprotective effects are unresolved, particularly constituent interactions with gastric ulcer-associated targets such as matrix metalloproteinase-9 (MMP9), a key mediator of mucosal degradation (Tarnawski et al., 2014; Dvornyk et al., 2021); while Das et al. (2012) demonstrated functional anti-ulcer activity *in vivo*, specific molecular targets and bioactive principles were not identified. Fourth, toxicological risk assessment of high-camphor genotypes is absent despite camphor’s narrow therapeutic index (minimum toxic dose: 50 mg/kg) and regulatory restrictions on oral consumption (FDA 21 CFR 310.545; Khine et al., 2009), raising safety concerns regarding breeding strategies maximizing camphor without exposure analysis. Furthermore, farmers and industries lack awareness regarding genotypic variation in bioactive content and its implications for breeding and pharmaceutical applications, while the species’ endangered status and absence of standardized cultivars necessitate urgent identification of elite germplasm combining superior agronomic performance with enhanced phytochemical profiles.

To address these gaps, the present study integrated comprehensive GC-MS/MS analysis followed by virtual screening and molecular dynamics simulations of black turmeric phytochemicals were conducted to computationally predict their potential gastroprotective mechanisms and generate testable hypotheses for subsequent biological validation. Farmers and industries often lack awareness about the characteristics of existing *C. caesia* germplasm, particularly concerning genotypic variation in bioactive content and its implications for breeding and pharmaceutical applications. Given the endangered status of this species and absence of standardized cultivars, there is an urgent need to identify elite genotypes combining superior agronomic performance with enhanced phytochemical profiles. With this backdrop, the objectives of the present study were to: (1) characterize morphological and phytochemical diversity among 21 black turmeric genotypes collected from diverse Indian agro-climatic zones, (2) quantify camphor content using validated GC-MS/MS methodology to identify high-camphor chemotypes, (3) evaluate genotype-environment interactions through multi-location trials, and (4) employ molecular docking and dynamics simulations to generate mechanistic hypotheses regarding potential gastroprotective properties that can direct future *in vitro* and *in vivo* validation studies.

## 2 Materials and methods

### 2.1 Collection of plant materials

**FIGURE 1:**
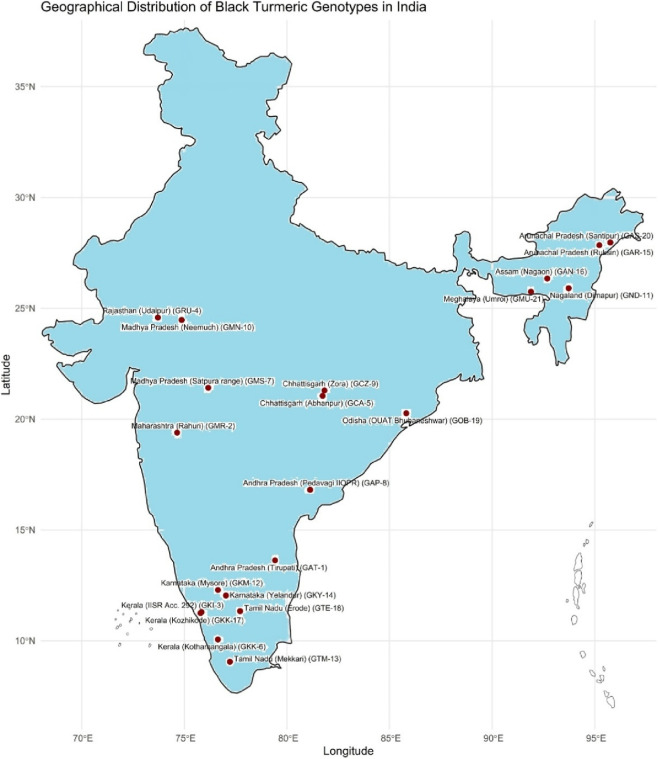
State-wise distribution and collection sites of *Curcuma caesia* genotypes across India.

The experimental material consisted of 21 genotypes of *C. caesia*, were collected from diverse agro-climatic regions across India to evaluate a wide range of genetic diversity. Rhizomes were sourced from multiple states and subsequently established under uniform cultivation conditions for evaluation. The details of their geographical origin are presented in Table 1 and Figure 1.

**TABLE 1:** Geographic origin of *Curcuma caesia* genotypes collected from different regions of India.

Genotypes	State	Geographical indication		Local name
		Latitude	Longitude	
GAT-1	Andhra Pradesh (tTirupati)	13.6288° N	79.4192° E	Nalla Pasupu
GMR-2	Maharashtra (Rahuri)	19.3951° N	74.6521° E	Kali Haldi
GKI-3	Kerala (IISR Acc. 292)	11.2985° N	75.8403° E	Kari Manjal
GRU-4	Rajasthan (Udaipur)	24.5854° N	73.7125° E	Kali Haldi
GCA-5	Chhattisgarh (Abhanpur)	21.0506° N	81.7400° E	Kali Haldi
GKK-6	Kerala (Kothamangala)	10.0603° N	76.6352° E	Kari Manjal
GMS-7	Madhya Pradesh (Satpura range)	21.4167° N	76.1667° E	Kali Haldi
GAP-8	Andhra Pradesh (Pedavagi IIOPR)	16.8121° N	81.1319° E	Nalla Pasupu
GCZ-9	Chhattisgarh (Zora)	21.2951° N	81.8282° E	Kali Haldi
GMN-10	Madhya Pradesh (Neemuch)	24.4738° N	74.8726° E	Kali Haldi
GND-11	Nagaland (Dimapur)	25.9091° N	93.7266° E	Kali Haldi
GKM-12	Karnataka (Mysore)	12.2958° N	76.6394° E	Kariarishina/karu arishina
GTM-13	Tamil Nadu (Mekkari)	9.0587° N	77.2215° E	Karumanjal
GKY-14	Karnataka (Yelandur)	12.0464° N	77.0279° E	Kariarishina/Naru Kachora
GAR-15	Arunachal Pradesh (Ruksin)	27.8489° N	95.2185° E	Yaingang Amuba
GAN-16	Assam (Nagaon)	26.3480° N	92.6838° E	Nar Kachur/Kali Haldi
GKK-17	Kerala (Kozhikode)	11.2488° N	75.7839° E	Kari Manjal
GTE-18	Tamil Nadu (Erode)	11.3410° N	77.7172° E	Karumanjal
GOB-19	Odisha (OUAT Bhubaneshwar)	20.2650° N	85.8117° E	Kala Haladi
GAS-20	Arunachal Pradesh (Santipur)	27.9684° N	95.7547° E	Kali Haldi
GMU-21	Meghalaya (Umroi)	25.7474° N	91.8890° E	Khasia Black Turmeric/Kala Haldhi

### 2.2 Experimental site and design

The experiment was conducted in two different locations from July 2024 to March 2025 at the Coconut Nursery Farm, Department of Spices and Plantation Crops, Horticultural College and Research Institute, Tamil Nadu Agricultural University, Coimbatore, India (Location 1) and Turmeric Research Centre, Bhavanisagar, TNAU, Erode (Location 2). It was situated at 11° 7′ N latitude, 77° 59′ E longitude, and at an altitude of 426 m above mean sea level (MSL) and 11° 29′N latitude and 77° 08′E longitude, with an altitude of 256 m above mean sea level (MSL). The experiment was laid out in Randomized Block Design (RBD) with two replications. Each black turmeric genotype was planted in two rows in a 3 m^2^ plot with a spacing of 30 cm × 30 cm. All the recommended practices, including fertilizer application and pest and disease management, were followed during the entire crop period.

### 2.3 Trait measurement

 Observations were recorded 90 days after sowing on key morphological traits including plant height, number of leaves per plant, leaf length, leaf width and days to maturity. Whereas, rhizomes were harvested at full maturity, to evaluate for yield traits such as length of rhizome (cm), girth of rhizome, number of rhizome plant^-1^, rhizome weight plant^-1^, rhizome yield plot^-1^ and essential oil yield according to the DUS guidelines as a measure of conservation of the species given by Protection of Plant Varieties and Farmers Rights Act (PPV&FR, 2009), Government of India. All the data of traits were statistically analyzed. Variability and diversity were computed by applying the Pearson correlation coefficient using SPSS 20.0 Version and R-Software. Heat map, dendrogram, and PCA were constructed by using R-Software.

### 2.4 Oil extraction and quantification

For oil yield, 100 gm of fresh rhizomes of each genotype. Were used for essential oil extraction by hydro-distillation method using a Clevenger apparatus. Rhizomes were sliced to a thickness of 2 mm or less and were transferred to 1-L short neck round bottom flask with 500 mL of water and boiled for 3 h (Lenka et al., 2025). After continuous running for 3 h and when no increment in essential oil quantity was visible for more than 1 h, the layer of essential oil was carefully separated. The water is removed from the oil by using a pinch of anhydrous sodium sulphate. The corresponding mild bluish purple coloured oil was recovered and calculated in terms of percentage (W/W). A method demonstrated to effectively isolate bioactive metabolites from spices plants (Paw et al.,2020) The oil was collected in Eppendorf tube and stored under 4 °C refrigeration to preserve metabolite integrity until analysis.

$$\text{Volatile oil}\left(\frac{w}{w}\right)\left(\%\right)=\frac{Amount\;of\;oil\;collected\;\left(mL\right)}{Weight\;of\;sample\;\left(mL\right)}✕\;100$$

### 2.5 Sample preparation for GC-MS analysis

For GC-MS analysis, 20 µL of each individual essential oil sample was re-dissolved in 0.1 mL of HPLC-grade hexane to ensure appropriate dilution. The mixture was vortexed thoroughly to ensure homogeneity and was injected into the GC-MS for analysis. This analytical approach aligns with recent advancements in phytochemical profiling techniques (Lenka et al., 2025).

### 2.6 Qualitative and quantitative analysis using gas chromatography-mass spectroscopy (GC-MS)

Phytochemical profiling of the *C. caesia* oil was conducted using a Shimadzu triple-quad GCMS-TQ™ 8040 NX system at the Central Instrumentation Laboratory, Centre of Plant Molecular Biology and Biotechnology, TNAU. The analysis utilized an Rxi-5MS capillary column (30 mm ID, 0.25 mm OD, 0.25 μm film thickness). Helium served as the carrier gas with a constant flow rate of 1.0 mL/min. Sample introduction employed a split mode injection (split ratio 1:10) with a 1 μL injection volume at 240 °C. The oven temperature program was optimized to enhance compound separation: initial temperature maintained at 70 °C for 15 min, followed by a programmed increase to 280 °C with a 3-min hold time. Detection parameters were configured as follows: interface temperature at 280 °C, ion source temperature at 230 °C, and mass spectrometric analysis conducted using Q3 scan mode with an m/z scanning range of 35–650.

D-camphor standard (Sigma-Aldrich, Product No. PHR1119) was used for camphor quantification, which was performed using a Shimadzu Nexis GC-2030, coupled with a Shimadzu GCMSTQ8040NX tandem mass spectrometer (Shimadzu, Kyoto, Japan). Camphor was separated with Shimadzu SH-Rxi-5Sil MS 30m, 0.25mm, 0.25m, 5M column. The flow rate of helium gas, 1 mL/min, was used as a carrier gas. The column oven temperature was programmed as follows: the initial temperature was 50 °C, held for 1 min, followed by an increase to 180 °C at a rate of 10 °C/min and held for 1 min. The analysis time was 18 min, including 3 min of equilibration time before each injection. The temperature of the injection port was maintained at 200 °C, and the injection volume was 1.0 µL with a split ratio value of 10. The temperature of the ion source and interface was 200 °C and 220 °C, respectively. The segmented MRM scan time was 8.5–9.5 min, as the camphor was eluted at a retention time of 8.95 min.

### 2.7 Molecular docking approach to identify antiulcer activity

#### 2.7.1 Selection of targets

Gastric ulcer target such as MMP9 (matrix metalloproteinase-9), IGFR (Insulin-like growth factor 1), EGFR (Epidermal Growth Factor Receptor) were chosen through a comprehensive literature survey, and their 3-dimensional X-ray crystal structure of MMP9 with PDB ID: 1ITV, IGFR with PDB ID: 5FXQ, EGFR with PDB ID: 1WT5 (Table 2) were obtained from the Protein Data Bank (https://www.rcsb.org/).

**TABLE 2:** Amino acids at binding interface of receptors.

Receptors	Amino acids at binding interface
1ITV (MMP9)	CYS A:4, ASN A:5, VAL A:6, ASN A:7, ILE A:8, PHE A:9, GLU A:14, ILE A:15, HIS A:150, ASP A:151, CYS A:162, ASP A:164, ARG A:165, PHE A:147, TYR A:20 TYR A:184, TYR A:458, TYR A:111, TYR A: 455, TYR A: 481,VAL A:185, THR A:186, TYR A:187, CYS A:192, PRO A:193, ASN A:177, LEU A: 67, ALA A: 159
5FXQ (IGFR)	PHE A:981, SER A:982, ALA A:983, ALA A:984, ASP A:985, VAL A:986, LEU A:1005, GLY A:1006, GLN A:1007, GLY A:1008, SER A:1009, PHE A:1010, GLY A:1011, VAL A:1013, ALA A:1031, LYS A:1033, THR A:1034, ILE A:1045, GLU A:1046, PHE A:1047, ASN A:1049, GLU A:1050, ALA A:1051, SER A:1052, VAL A:1053, MET A:1054, LYS A:1055, GLU A:1056, VAL A:1063, MET A:1079, GLU A:1080, LEU A:1081, MET A:1082, GLY A:1085, ASP A:1086, LEU A:1126, ASN A:1129, LYS A:1130, PHE A:1131, VAL A:1013, HIS A:1133, ARG A:1134, ASP A:1135, ARG A:1139, ASN A:1140, CYS A:1141, MET A:1142, GLY A:1152, ASP A:1153, PHE A:1154, GLY A:1155, MET A:1156, THR A:1157, ARG A:1158, ASP A:1159, ILE A:1160, TYR A:1161
1WT5 (EGFR)	SER A: 25, GLN A: 39, GLU A: 40, ILE A: 76, GLY A:44, ILE A: 76, THR A: 78, ALA A: 92, TYR A: 95, TYR A: 111, GLY A:109, GLN A: 110, LEU A: 113, ARG A:169, TYR A:160, LEU A:188, TYR A: 458, TRP A: 477, TYR A: 481

#### 2.7.2 Protein and ligand preparation

The gas chromatography coupled with mass spectrometry (GC-MS) revealed the presence of a total of 66 metabolites in black turmeric. After SWISS ADME screening, 38 phytochemicals were identified, and the ligands library was constructed by generating the 2-dimensional structure of the metabolites using the PubChem database (https://pubchem.ncbi.nlm.nih.gov/) in SDF format. In preparation for molecular docking studies, both the target protein and selected metabolites underwent essential pre-processing using BIOVIA Discovery Studio Visualizer (DS 4.5, Accelrys, Inc., San Diego, CA, United States). The receptor protein was prepared via the Macromolecule module, which included energy minimization and the addition of protonation sites to ensure proper structural integrity. Protein energy minimization was carried out using the CHARMm force field, with the root mean square deviation (RMSD) maintained at 0.25 Å. Ligands were also energy minimized and prepared for docking using the small molecule protocol. To accurately simulate 3D structures of the metabolites, parameters for ionization, tautomerization, and isomer generation were set to default settings, facilitating reliable docking simulations. The pharmacokinetic properties of these metabolites were evaluated using the SWISS-ADME web tool (http://www.swissadme.ch/), and only those metabolites that met the drug-likeness criteria were chosen for further docking analysis. Boiled Egg model was applied to assess the potential of these metabolites to cross the blood-brain barrier and their absorption through the gastrointestinal tract. This analysis is a key component in early drug discovery, helping researchers evaluate whether a metabolites is a viable therapeutic candidate (Daina et al., 2017).

#### 2.7.3 Prediction of binding sites

The active sites for the targets were predicted by the Computed Atlas of Surface Topography of Proteins (CASTp) online server (http://sts.bioe.uic.edu/castp/) (Tian et al., 2018) and BIOVIA Discovery Studio (DS4.5, Accelrys, Inc., San Diego, CA, United States) (Accelrys [2.1], 2008).

#### 2.7.4 Molecular docking analysis and virtual screening

The target protein was prepared by removing the water molecules and other cofactors before docking the ligands into the protein’s binding site. The gastric ulcer target proteins (PDB ID: 1ITV, 5FXQ, 1WT5) were then imported into PyRx 0.8. Before docking, macromolecules of the target protein were obtained in PyRx 0.8 using the Autodock Vina module that generates the PDBQT of targets. A grid box was generated around the binding pocket of the targets on the x, y, and z-axes to perform molecular docking Table 3. The bioactive metabolite from *C. caesia* was used as the ligand. The 2D structure of metabolite were retrieved from the PubChem database. The PDB structure of metabolites was converted to PDBQT format, and the energy was minimized using PyRx 0.8. The same steps were performed for standard drug Ranitidine. The BIOVIA Discovery Studio Visualizer was used to visualize the interaction between gastric ulcer targets and bioactive metabolites from *C. caesia* with standard drug, enabling detailed examination of amino acid interactions at the active sites. This comprehensive analysis identified specific binding patterns, hydrogen bonds, and other non-covalent interactions contributing to binding affinity, facilitating the identification of the most promising *C. caesia* metabolites with potential -targeting properties.

**TABLE 3:** X, Y, and Z coordinates and dimensions of gastric ulcer target binding sites.

S.NO	Target protein	PDB ID	Coordinates			Dimensions		
​	​	​	X	Y	Z	X	Y	Z
1	MMP9	1ITV	42.052	30.858	7.2647	46.2923	56.6168	35.2550
2	IGFR	5FXQ	22.8749	15.4964	46.2954	84.6636	60.9155	59.1265
3	EGFR	6O19	36.8621	23.8942	42.0135	60.0940	36.8368	51.1480

#### 2.7.5 Molecular dynamic simulation

Molecular dynamics (MD) simulations of the 1ITV-Camphor complex were performed using the GROMACS 4.5.3 software suite, employing the CHARMM36 force field (Hess et al., 2008) over a 100-nanosecond (ns) simulation period. Initial system preparation involved generating topology files from the Protein Data Bank (PDB) structure using ‘pdb2gmx’ and the CGenFF server for ligand parameterization. The system was subsequently solvated in an TIP3 solvated water model via the ‘genbox’ utility, followed by ion addition using ‘genion’ to achieve system charge neutrality. Prior to production dynamics, energy minimization was conducted using the steepest descent algorithm to eliminate steric clashes and optimize system stability, facilitated by pre-processing with ‘grompp’ and execution via ‘mdrun’. Subsequently, the system underwent equilibration through a two-step protocol: first under NVT (canonical ensemble) conditions, followed by NPT (isothermal-isobaric ensemble) conditions, each spanning 100 picoseconds (ps) at a physiological temperature of 310 K. During equilibration, positional restraints were applied to the ligand, and temperature coupling groups were implemented to ensure proper thermalization. Production MD simulations were executed following parameter configuration via ‘grompp’ and trajectory generation using ‘mdrun’. Post-simulation, trajectory processing included structural recentering within the unit cell using ‘trjconv’. Trajectory analysis was performed using GROMACS-integrated tools to derive thermodynamic and dynamic properties, while data visualization was conducted using XMGRACE.

## 3 Results

### 3.1 Performance of black turmeric genotype on the qualitative traits based on DUS scoring

**FIGURE 2:**
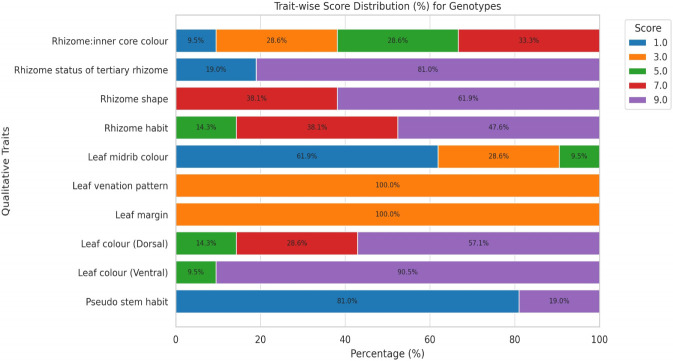
Grouping of 21 genotypes qualitative characters through DUS guidelines.

The qualitative assessment of 21 *C. caesia* genotypes following DUS guidelines revealed significant morphological variation across stem, leaf, and rhizome characters (Supplementary Table S1; Table 4; Figure 2). Pseudo stem habit exhibited clear dimorphism: 81.0% of genotypes (17 genotypes) displayed compact growth, while 19.0% (4 genotypes: GMS-7, GND-11, GTM-13, GMU-21) showed open type.

**TABLE 4:** DUS scoring of the qualitative characters for the 21 genotypes.

Characters	Pseudo stem habit	Leaf colour (ventral)	Leaf colour (dorsal)	Leaf margin	Leaf venation pattern	Leaf midrib colour	Rhizome habit	Rhizome shape	Rhizome status of tertiary rhizome	Rhizome: Inner core colour
Genotypes										
GAT-1	1	5	5	3	3	1	5	5	1	7
GMR-2	1	5	3	3	3	3	3	5	9	3
GKI-3	1	5	5	3	3	1	5	5	9	5
GRU-4	1	5	3	3	3	5	3	5	9	3
GCA-5	1	5	5	3	3	1	5	3	9	5
GKK-6	1	5	3	3	3	1	3	3	9	3
GMS-7	9	7	7	3	3	1	5	3	9	7
GAP-8	1	5	5	3	3	1	5	5	9	7
GCZ-9	1	5	5	3	3	3	5	5	1	7
GMN-10	1	5	5	3	3	3	7	5	9	1
GND-11	9	5	5	3	3	1	3	5	9	7
GKM-12	1	5	3	3	3	3	3	3	9	7
GTM-13	9	5	5	3	3	1	3	3	9	5
GKY-14	1	5	5	3	3	1	5	3	1	1
GAR-15	1	5	5	3	3	3	7	3	9	3
GAN-16	1	5	3	3	3	5	3	3	9	5
GKK-17	1	5	5	3	3	1	5	5	9	3
GTE-18	1	5	5	3	3	1	5	5	9	3
GOB-19	1	7	7	3	3	1	3	5	1	5
GAS-20	1	5	7	3	3	1	5	5	9	5
GMU-21	9	5	3	3	3	3	7	5	9	3

Leaf characteristics demonstrated both dimorphic and polymorphic patterns. Ventral leaf color was predominantly green in 90.5% of genotypes (19 accessions), with only GMS-7 and GOB-19 exhibiting dark green coloration. Dorsal leaf color showed greater polymorphism: 57.1% green (12 genotypes), 28.6% light green (6 genotypes), and 14.3% dark green (3 genotypes). Leaf margin and venation pattern were monomorphic, with all genotypes displaying even margins and closely spaced venation. Leaf midrib color revealed distinct variation: 61.9% violet-blue group (13 genotypes), 28.6% purple group (6 genotypes), and 9.5% greyed purple group (2 genotypes: GRU-4, GAN-16).

Rhizome characteristics showed the most diagnostic variation. Rhizome habit was polymorphic: 38.1% compact (8 genotypes), 47.6% intermediate (10 genotypes), and 14.3% loose (3 genotypes: GMN-10, GAR-15, GMU-21). Rhizome shape exhibited dimorphism with 61.9% curved (13 genotypes) and 38.1% straight (8 genotypes). Tertiary rhizomes were present in 81.0% of genotypes and absent in 19.0% (GAT-1, GCZ-9, GKY-14, GOB-19). Rhizome inner core color displayed high polymorphism: 33.3% moderate blue (7 genotypes), 28.6% each for strong blue and greenish blue (6 genotypes each), and 9.5% light blue (2 genotypes: GMN-10, GKY-14).

### 3.2 Quantitative trait performance at location 1 (Coimbatore)

At Coimbatore, plant height ranged from 27.81 cm (GAR-15) to 69.09 cm (GMN-10) with a mean of 44.46 cm and CV of 22.68% (Table 5; Supplementary Table S2). Eleven genotypes exceeded the population mean. Number of leaves ranged from 4.00 (GAT-1) to 5.30 (GAP-8, GKY-14) with a mean of 4.79 leaves. Leaf length varied from 16.50 cm (GAR-15) to 22.64 cm (GMN-10), averaging 19.92 cm. Leaf width ranged from 8.84 cm (GMN-10) to 12.61 cm (GKY-14) with mean 10.42 cm. Days to maturity ranged narrowly from 237.50 (GAN-16) to 244.00 (GND-11) days, averaging 240.71 days with low CV (0.79%), indicating uniform crop duration. Rhizome length varied from 4.42 cm (GAS-20) to 6.72 cm (GAN-16), averaging 5.83 cm. Rhizome girth ranged from 5.61 cm (GTM-13) to 9.01 cm (GMN-10) with mean 7.42 cm. Number of rhizomes per plant ranged from 2.35 (GAT-1) to 4.45 (GTE-18), averaging 3.59. Rhizome weight per plant varied from 68.80 g (GTM-13) to 88.87 g (GMR-2) with mean 76.08 g and CV 6.75%. Rhizome yield per plot ranged from 0.66 kg (GAP-8) to 0.92 kg (GMR-2), averaging 0.80 kg. Essential oil yield showed substantial variation: 0.20% (GMS-7, GAR-15) to 0.40% (GMR-2, GMN-10, GTE-18) with mean 0.29% and high CV (19.72%).

**TABLE 5:** Descriptive statistical analysis for quantitative traits among 21 black turmeric genotypes in location 1 at Coimbatore.

S.No	Traits	Mean	Range		CV (%)	Cd (0.05)	PCV	Gcv	H^2^	GA	GAM
			Min	Max							
1	PH	44.46 ± 10.08	27.81	69.09	22.68	6.49	22.72	22.64	99.34	20.67	46.49
2	NL	4.79 ± 0.32	4.00	5.30	6.66	0.20	8.51	4.04	22.52	0.19	3.95
3	LL	19.92 ± 1.59	16.50	22.64	7.98	1.02	9.76	5.67	33.69	1.35	6.77
4	LW	10.42 ± 1.02	8.84	12.61	9.82	0.66	14.32	1.80	1.57	0.05	0.46
5	DM	240.79 ± 2.02	237.40	244.00	0.79	1.22	0.90	0.77	74.26	3.31	1.37
6	LR	5.83 ± 0.55	4.42	6.72	9.51	0.36	13.97	4.48	10.30	0.18	2.96
7	GR	7.42 ± 0.80	5.61	9.01	10.77	0.51	13.21	7.56	32.79	0.66	8.92
8	NRPP	3.59 ± 0.56	2.40	3.60	15.52	0.36	13.64	5.97	19.12	0.17	5.37
9	RWPP	76.07 ± 5.13	68.80	88.87	6.75	3.30	9.27	2.28	6.04	0.88	1.15
10	RYPP	0.80 ± 0.07	0.66	0.92	9.35	0.05	12.40	4.20	11.46	0.02	2.93
11	EO	0.29 ± 0.06	0.20	0.40	19.72	0.04	28.79	4.13	2.06	0.00	1.22

Heritability estimates revealed high genetic control for plant height (H^2^ = 99.34%) and days to maturity (H^2^ = 74.26%). Moderate heritability was observed for leaf length (H^2^ = 33.69%) and rhizome girth (H^2^ = 32.79%).

### 3.3 Quantitative trait performance at location 2 (Bhavanisagar)

At Bhavanisagar, plant height ranged from 77.71 cm (GAR-15) to 107.08 cm (GKY-14) with mean 82.93 cm and reduced CV (9.72%) compared to Coimbatore (Table 6; Supplementary Table S3). Number of leaves showed greater variation: 3.90 (GOB-19) to 7.90 (GMN-10, GAP-8) with mean 6.53 and CV 22.24%. Leaf length ranged from 34.40 cm (GCA-5) to 50.05 cm (GAP-8), averaging 42.37 cm. Leaf width varied from 8.22 cm (GOB-19) to 18.01 cm (GTM-13) with mean 12.68 cm. Days to maturity ranged from 240.00 (GMR-2) to 249.50 (GMR-2) with mean 242.69 days. Rhizome length varied from 6.09 cm (GTM-13) to 9.50 cm (GMN-10), averaging 7.74 cm. Rhizome girth ranged from 6.79 cm (GAR-15) to 12.02 cm (GAN-16) with mean 10.20 cm. Number of rhizomes per plant ranged from 3.60 (GAR-15) to 5.50 (GCA-5), averaging 5.01. Rhizome weight per plant showed dramatic variation: 135.56 g (GAR-15) to 202.13 g (GMN-10) with mean 179.36 g, representing a 135.8% increase over Coimbatore. Rhizome yield per plot ranged from 1.19 kg (GKI-3) to 1.92 kg (GMN-10) with mean 1.58 kg. Essential oil yield ranged from 0.44% (GMS-7, GAR-15) to 0.50% (GMN-10) with mean 0.48%.

**TABLE 6:** Descriptive statistical analysis for quantitative traits among 21 black turmeric genotypes in location 2 at Bhavanisagar.

S.No	Traits	Mean	Range		CV (%)	Cd (0.05)	PCV	Gcv	H^2^	GA	GAM
			Min	Max							
1	PH	82.95 ± 8.06	77.71	107.08	22.24	5.19	9.91	9.76	96.99	16.42	19.79
2	NL	6.53 ± 1.45	3.90	7.90	9.72	0.94	22.73	21.73	91.34	2.79	42.78
3	LL	42.37 ± 6.42	34.40	50.05	24.08	4.13	15.53	14.76	90.30	12.24	28.89
4	LW	12.68 ± 3.05	8.22	18.01	15.15	1.96	24.34	23.83	95.87	6.09	48.07
5	DM	242.69 ± 2.26	240.00	249.50	0.93	1.45	0.95	0.77	66.45	3.15	1.30
6	LR	7.74 ± 0.76	6.09	9.50	9.76	0.49	19.79	1.68	0.72	0.00	0.29
7	GR	10.20 ± 1.40	6.79	12.02	13.71	0.90	16.86	9.60	32.44	1.15	11.27
8	NRPP	5.01 ± 0.44	3.60	5.50	8.86	0.29	9.51	8.15	73.45	0.72	14.39
9	RWPP	179.36 ± 18.03	135.56	202.13	10.05	11.61	23.42	21.90	87.47	56.90	42.20
10	RYPP	1.58 ± 0.22	1.19	1.92	12.89	0.13	19.79	1.68	0.72	0.00	0.29
11	EO	0.48 ± 0.03	0.44	0.50	7.06	0.02	9.98	4.87	23.81	0.02	4.89

High heritability was maintained for plant height (H^2^ = 96.99%), leaf width (H^2^ = 95.87%), number of leaves (H^2^ = 91.34%), leaf length (H^2^ = 90.30%), leaf width (H^2^ = 95.87%), and rhizome weight (H^2^ = 87.47%).

### 3.4 GC-MS analysis

**FIGURE 3:**
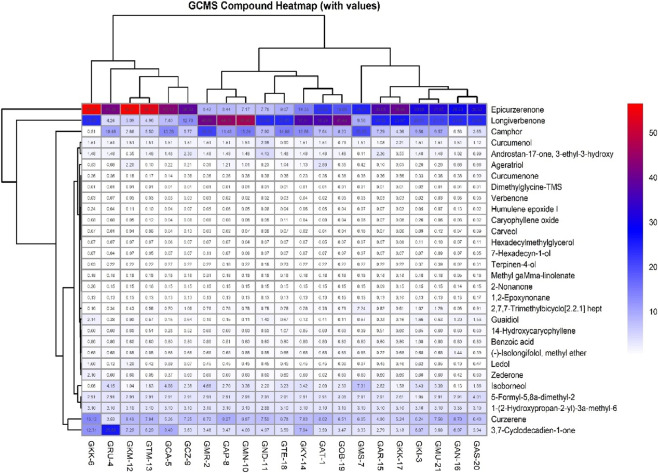
Heat-map chart of bioactive contents among 21 black turmeric genotypes.

GC–MS analysis identified 66 metabolites in *C. caesia* rhizome extracts across a retention time window of 7.8–30.5 min. Thirty metabolites with highest relative abundance were characterized using NIST20 spectral library (Supplementary Table S4; Figure 3). Major metabolite classes included monoterpenes (camphor, isoborneol, terpinen-4-ol), sesquiterpenes (curzerene, epicurzerenone, longiverbenone, curcumenone), and oxygenated derivatives. Camphor content varied substantially across genotypes: 0.51% (GKK-6) to 20.91% (GMR-2) in semi-quantitative GC–MS analysis. High-camphor genotypes included GMR-2 (20.91%), GMS-7 (20.20%), GMN-10 (15.24%), GTE-18 (14.60%), GCA-5 (13.28%), and GKY-14 (12.68%). Low-camphor genotypes included GKK-6 (0.51%), GAN-16 (0.58%), GKM-12 (2.86%), and GAS-20 (3.65%). Epicurzerenone showed inverse distribution: GKK-6 (56.44%), GKM-12 (56.27%), GTM-13 (54.13%), GCA-5 (43.10%), and GRU-4 (41.38%) exhibited highest concentrations, while GMR-2 (8.42%), GMN-10 (7.17%), GND-11 (7.78%), and GTE-18 (9.07%) showed lowest levels. Curzerene content was relatively uniform: 3.53% (GRU-4) to 13.12% (GKK-6), with most genotypes ranging 6%–9%. Longiverbenone was abundant in GAP-8 (44.59%), GMN-10 (46.40%), GOB-19 (40.44%), GMR-2 (40.66%), and GKY-14 (37.01%), but absent in several genotypes (GKK-6, GAN-16, GND-11, GTE-18).

### 3.5 Camphor quantification using GC-MS/MS

**FIGURE 4:**
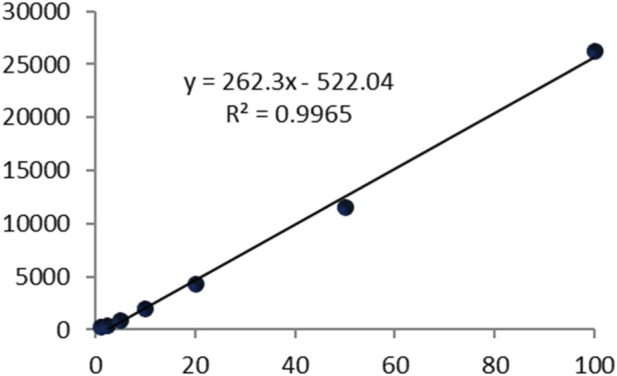
Calibration curve of camphor under the developed method from 1 to 500 μg/mL.

**FIGURE 5:**
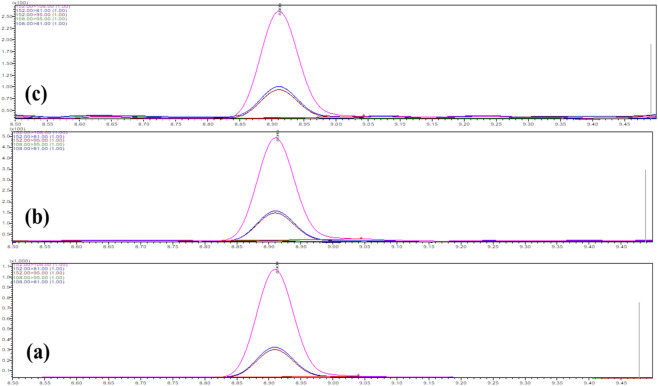
**(a)** Chromatogram of LOD level camphor 0.3 μg/mL, **(b)** LOQ Level camphor 1 μg/mL **(c)** camphor extracted from oil sample.

This study presents the first report on the quantitative estimation of camphor content in *C. caesia* rhizomes using a validated gas chromatography–mass spectrometry (GC–MS/MS) method. A Calibration curve analysis using D-camphor standard yielded linear regression equation y = 262.3x − 522.04 with excellent correlation (*R* ^2^ = 0.9965) over concentration range 1–500 μg/mL (Figure 4). Limit of detection (LOD) was 0.3 μg/mL and limit of quantification (LOQ) was 1 μg/mL. Chromatograms showed well-resolved, symmetrical camphor peaks at retention time 8.95 min without interfering signals (Figure 5). Validated quantification revealed substantial genotypic variation in camphor content (Table 7), 5.19% (GKK-6) to 33.84% (GTE-18), representing 6.5-fold difference. High-camphor genotypes included GTE-18 (33.84%), GMR-2 (30.35%), GMN-10 (26.41%), GCA-5 (24.44%), GRU-4 (23.32%), GKY-14 (22.18%), and GAP-8 (21.78%). Intermediate camphor genotypes included GMS-7 (19.07%), GOB-19 (18.03%), GKI-3 (16.34%), GMU-21 (16.34%), GND-11 (15.93%), and GAT-1 (14.09%). Low-camphor genotypes included GAR-15 (13.68%), GKK-17 (11.23%), GCZ-9 (11.01%), GKM-12 (9.39%), GAS-20 (7.61%), GTM-13 (7.00%), GAN-16 (5.20%), and GKK-6 (5.19%).

**TABLE 7:** Confirmative analysis of camphor content from 21 genotype through GC-MS/MS.

Sample name	Conc. %
GAT-1- Andhra Pradesh (Tirupati)	14.09
GMR-2- Maharashtra (Rahuri)	30.35
GKI-3- Kerala (IISR Acc. 292)	16.34
GRU-4- Rajasthan (Udaipur)	23.32
GCA-5- Chhattisgarh (Abhanpur)	24.44
GKK-6- Kerala (Kothamangala)	5.19
GMS-7- Madhya Pradesh (Satpura range)	19.07
GAP-8- Andhra Pradesh (Pedavagi IIOPR)	21.78
GCZ-9- Chhattisgarh (Zora)	11.01
GMN-10- Madhya Pradesh (Neemuch)	26.41
GND-11- Nagaland (Dimapur)	15.93
GKM-12- Karnataka (Mysore)	9.39
GTM-13- Tamil Nadu (Mekkari)	7.00
GKY-14- Karnataka (Yelandur)	22.18
GAR-15- Arunachal Pradesh (Ruksin)	13.68
GAN-16- Assam (Nagaon)	5.20
GKK-17- Kerala (Kozhikode)	11.23
GTE-18- Tamil Nadu (Erode)	33.84
GOB-19- Odisha (OUAT Bhubaneshwar)	18.03
GAS-20- Arunachal Pradesh (Santipur)	7.61
GMU-21- Meghalaya (Umroi)	16.34

### 3.6 *In silico* docking study

**FIGURE 6:**
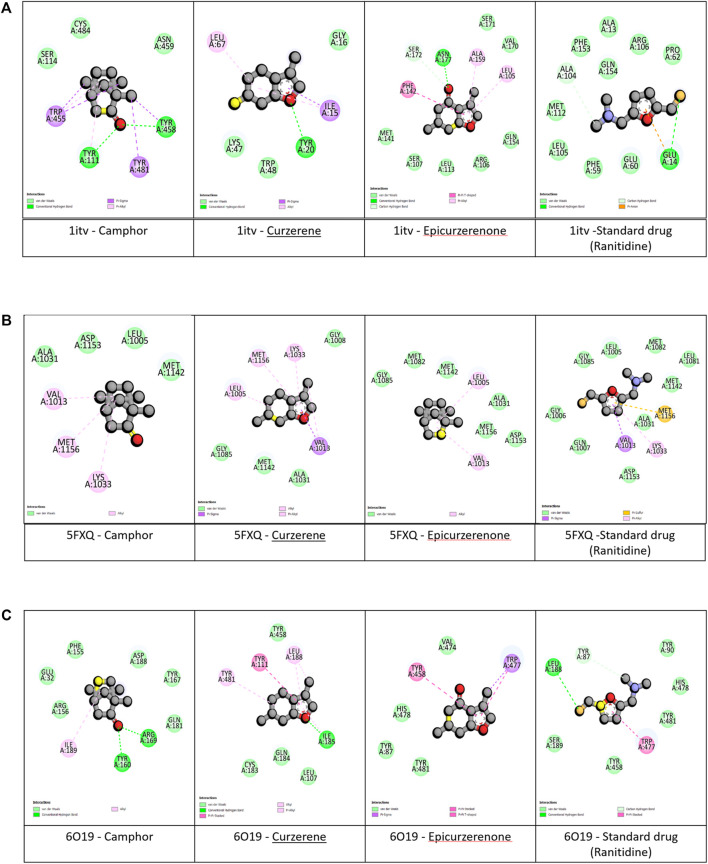
**(A)** 2D interaction plot of top three phytochemicals and standard drug with MMP9 (1itv) complexes. **(B)** 2D interaction plot of top three phytochemicals and standard drug with IGFR (5fxq) complexes. **(C)** 2D interaction plot of top three phytochemicals and standard drug with (1WT5) complexes.

**FIGURE 7:**
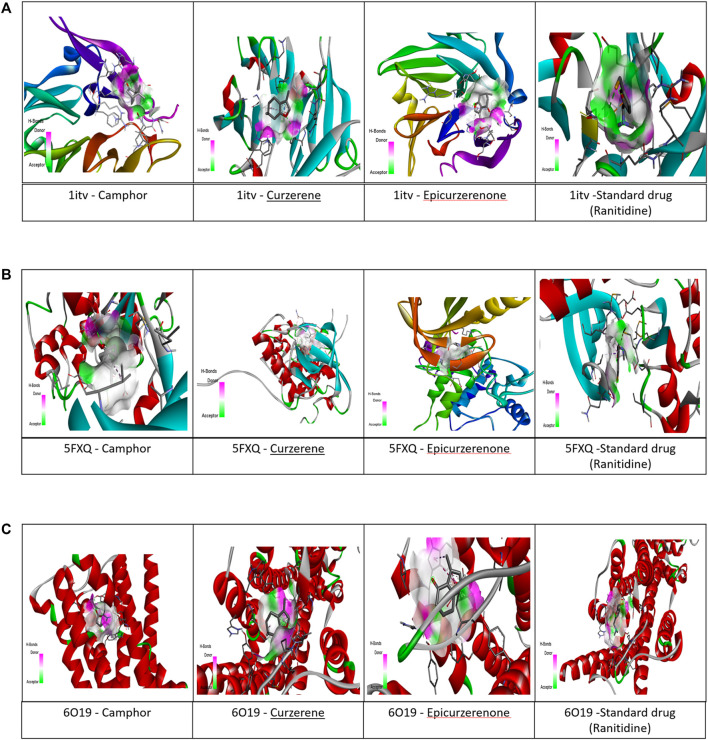
**(A)** 3D interaction plot of top three phytochemicals and standard drug with MMP9 (1itv) complexes. **(B)** 3D interaction plot of top three phytochemicals and standard drug with IGFR (5fxq) complexes. **(C)** 3D interaction plot of top three phytochemicals and standard drug with (1WT5) complexes.

The selected metabolite such as camphor, curzerene, epicurzerenone and standard drug (Ranitidine) (Elshamy et al., 2020) underwent virtual screening, targeting antiulcer receptors such as MMP9, IGFR, EGFR. The selected receptors, namely, 1ITV, 5FXQ, and 1WT5, are associated with gastric protective functions such as mucosal healing, inflammation regulation, and oxidative stress response. Among the metabolites tested, camphor exhibited the highest predicted binding affinity with 1ITV (Figure 6A; Figure 7A) (−7.8 kcal/mol), compared to the standard drug - Ranitidne (−6.3 kcal/mol) Table 8. The interaction was stabilized through hydrogen bonding interactions with key residues such as TYR A:111, TYR A:458. Furthermore, hydrophobic interactions were present in the form of Van der waals, Pi-Sigma and Pi-alkyl bonds with TRP A:455 and TYR A:481. Curzerene, on the other hand, showed the highest binding affinity with 5FXQ (Figure 6B; Figure 7B) (−6.8 kcal/mol), with important interactions involving GLY A:1,085, MET A:1,142, ALA A:1,031, GLY A:1,008, VAL A:1,013, LEU A:1,005, MET A:1,156 and LYS A:1,033, forming hydrophobic and Van der Waals interactions within the binding pocket. For 1WT5 (Figure 6C; Figure 7C), curzerene again demonstrated strong binding (−7.1 kcal/mol), primarily interacting with residues like ILE A:185, TYR A: 481, TYR A: 111, LEU A: 188, which contribute to its receptor stability. Epicurzerenone also showed moderate binding affinities across all three receptors (−5.1 to −6.7 kcal/mol), forming interactions with residues such as ILE A:56, SER A:54, and ALA A:57. Although slightly lower in affinity compared to camphor and curzerene, epicurzerenone maintained consistent receptor engagement, suggesting its supportive therapeutic role. Overall, computational modeling predicts that camphor and curzerene form favorable interactions with key active site residues of gastric ulcer-associated proteins, suggesting potential for further investigation as natural gastroprotective metabolites. However, these *in silico* predictions represent preliminary screening results that require experimental validation. Based on highest docking scores and strong receptor-binding profiles compared to standard drugs, camphor emerges as the priority lead metabolite for subsequent *in vitro* enzyme inhibition assays (e.g., MMP9 zymography) and cellular cytoprotection studies to determine whether predicted binding affinity translates to functional gastroprotective activity.

**TABLE 8:** Docking result of six anti-ulcer receptors with the highest binding affinity ligands of camphor.

Protein	CID	Name	Binding affinity	No. of H-Bond	Interacting active site residue
1ITV	2537	Camphor	−7.8	2	TYR A:458, TYR A:111, TYR A: 455, TYR A: 481
	12305301	Curzerene	−4.6	1	TYR A:20, LEU A: 67, ILE A: 15
	5317062	Epicurzerenone	−5.1	1	ASN A:177, PHE A: 147, ALA A: 159
	3001055	Standard drug (Ranitidine)	−6.3	1	GLU A:14
5FXQ	2537	Camphor	−5.2	0	VAL A: 1013, MET A: 1156, LYS A: 1033
	12305301	Curzerene	−6.8	0	VAL A: 1013, MET A: 1156, LYS A: 1033, LEU A: 1005
	5317062	Epicurzerenone	−6.7	0	VAL A: 1013, LEU A: 1005
	3001055	Standard drug (Ranitidine)	−4.8	0	VAL A: 1013, MET A: 1156, LYS A: 1033
1WT5	2537	Camphor	−6.8	2	ARG A:169, TYR A:160, ILE A: 189
	12305301	Curzerene	−7.1	1	ILE A:185, TYR A: 481, TYR A: 111, LEU A: 188
	5317062	Epicurzerenone	−6.7	0	TRP A: 477, TYR A: 458
	3001055	Standard drug (Ranitidine)	−5.5	1	LEU A:188, TRP A: 477

### 3.7 Molecular dynamics simulation

**FIGURE 8:**
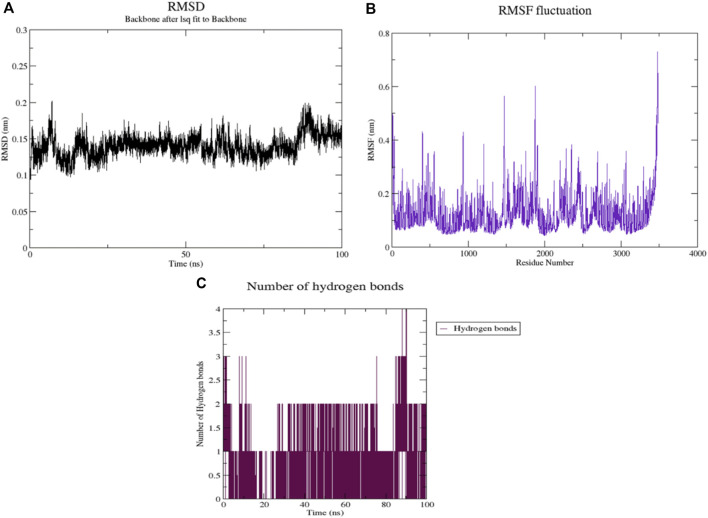
**(A)** Root mean square deviation for MMP9- camphor complex, **(B)** Root Mean square fluctuations MMP9 - camphor complex, **(C)** Number of hydrogen bonds.

The optimal docked complex of MMP9 with camphor was subjected to a 100 ns molecular dynamics (MD) simulation to assess the structural stability and conformational behavior of the protein-ligand complex. The Root Mean Square Deviation (RMSD) plot, which represents the average displacement of the protein backbone atoms over time, was analyzed to evaluate the system’s equilibration and structural integrity. At the start of the simulation, the backbone RMSD begins at around 0.10 nm, indicating a stable initial conformation. Over the first 20–30 ns, the RMSD gradually increases and fluctuates between 0.13 and 0.17 nm, suggesting minor conformational rearrangements as the protein to the ligand binding. Between 30 and 80 ns, the system maintains an overall stable RMSD with moderate fluctuations, remaining consistently between 0.22 and 0.28 nm, indicative of equilibrium in the complex. Toward the end of the simulation, from 80 to 100 ns, a slight decrease in RMSD is observed, falling back to around 0.20 nm, suggesting a re-stabilization of the protein-ligand conformation. The overall RMSD trend indicates that MMP9 –camphor complex achieves conformational stability after initial adjustments, and no major structural distortions were observed throughout the 100 ns trajectory. The relatively low average RMSD values (0.20–0.25 nm) confirm that camphor remains stably bound within the MMP9 binding pocket, it shows MMP9 with camphor have properties of gastric ulcer treatment (Figure 8A).

The Root Mean Square Fluctuation (RMSF) values indicate that certain regions of the receptor remain stable, with fluctuations around 0.1–0.2nm, suggesting minimal movement throughout the simulation, as shown in Figure 8B. Notably, peaks with RMSF values between 0.5 and 0.7 nm point to highly flexible regions, likely corresponding to loop areas, flexible termini, or binding sites crucial for receptor function.

The interaction of hydrogen bonds is essential for maintaining the stability of the complex. The hydrogen bond analysis depicted in Figure 8C revealed two key interactions over the course of the simulation. One hydrogen bond persisted throughout the simulation, though with periodic interruptions, indicating fluctuations in its stability. The second hydrogen bond was transient, forming only for brief intervals during the simulation, contributing minimally to the overall stability of the complex.

This observation implies that the receptor-docked complex remains structurally stable, with only minor fluctuations typical of natural molecular dynamics, and does not undergo any substantial conformational changes.

### 3.8 *In vitro* studies supporting gastroprotective mechanisms

Curcuma caesia rhizome essential oil has demonstrated significant anti-inflammatory activity relevant to gastric ulcer pathophysiology, as evidenced by Paw et al. (2020) who evaluated essential oil (0.5%–2.0% v/v; composition: camphor 28.6%, curzerene 12.4%, ar-turmerone 8.9%) in carrageenan-induced rat paw edema and *in vitro* nitric oxide scavenging assays. The essential oil exhibited 62.4% paw edema inhibition at the 3-h timepoint, comparable to the standard anti-inflammatory drug indomethacin (73.8%), with an *in vitro* nitric oxide scavenging IC_50_ of 48.3 μg/mL and 54% downregulation of COX-2 expression in LPS-stimulated macrophages assessed by Western blot analysis (Paw et al., 2020). These anti-inflammatory effects are directly relevant to gastric ulcer pathogenesis, as inflammation contributes to mucosal damage through COX-2-mediated prostaglandin dysregulation, neutrophil infiltration, and oxidative stress, suggesting that *C. caesia* may exert gastroprotective effects through dual mechanisms: reducing inflammatory damage while potentially preserving cytoprotective prostaglandin synthesis *via* the COX-1 pathway. Additionally, Liu et al. (2013) demonstrated robust antioxidant activity through DPPH radical scavenging (IC_50_ = 32.8 μg/mL), ABTS radical scavenging (IC_50_ = 28.4 μg/mL), and lipid peroxidation inhibition (76.8% at 100 μg/mL), which is critical for preventing oxidative stress-induced gastric mucosal damage, while Angel et al. (2012) reported antibacterial activity against Gram-negative pathogens (*Escherichia coli, Klebsiella pneumoniae*) with zones of inhibition ranging 16.2–18.4 mm and minimum inhibitory concentrations of 125–250 μg/mL, suggesting potential activity against *Helicobacter pylori*, the causative agent in 60%–90% of peptic ulcer cases, though direct anti-*Helicobacter* studies remain to be conducted.

### 3.9 Toxicological safety assessment

The identification of genotypes with exceptionally high camphor content—particularly GTE-18 (33.84%), GMR-2 (30.35%), and GMN-10 (26.41%)—necessitates critical evaluation of safety implications, as camphor exhibits a narrow therapeutic index with well-documented neurotoxic and hepatotoxic effects at doses marginally exceeding therapeutic levels (Khine et al., 2009; Love et al., 2004). Quantitative risk assessment of traditional consumption patterns (2 g/day rhizome powder; Rajkumari and Sanatombi, 2017) revealed that even the highest-camphor genotype GTE-18 delivers only 4.67 mg camphor per dose (0.078 mg/kg for 60 kg adults), representing 3.9% of the WHO daily intake limit (2 mg/kg/day; World Health Organization, 1999) and maintaining a 641-fold safety margin below minimum toxic doses (50 mg/kg; Khine et al., 2009), indicating acceptable safety profiles for adult populations at conventional dosing. However, pediatric populations exhibit three to five fold greater sensitivity to camphor-induced seizures, with toxicity reported at exposures as low as 30–50 mg (Love et al., 2004), warranting explicit contraindications for high-camphor genotypes (>25%) in children under 12 years, consistent with European Medicines Agency guidelines (EMA, 2012). These findings necessitate application-specific breeding strategies rather than uniform camphor maximization: moderate-camphor genotypes (10%–20% camphor; GAT-1, GMS-7, GOB-19) are recommended for direct traditional use, offering enhanced safety margins suitable for chronic consumption and broader patient populations; medium-high camphor genotypes (20%–30%; GMN-10, GCA-5, GRU-4) for pharmaceutical standardization enabling precise dose control; and extreme high-camphor genotypes (>30%; GTE-18, GMR-2) exclusively for industrial camphor isolation or formulation-controlled application.

## 4 Discussion

The evaluation of genetic variation provides critical information about the uniqueness and distinctness of germplasm accessions, which is fundamental for effective conservation, utilization, and crop improvement programs. The morphological characterization demonstrated pronounced variability in vegetative and rhizome traits, particularly pseudostem architecture and rhizome pigmentation patterns, confirming the rich genetic foundation of this endangered species. Such phenotypic diversity typically reflects genotypic adaptation to heterogeneous environments, a phenomenon well-documented in related *Curcuma* species where morphological variation correlates closely with phytochemical diversity and environmental responsiveness (Mahanta et al., 2023; Benya et al., 2023). These findings align with previous reports on *Curcuma* species, emphasizing the importance of qualitative trait diversity in genetic improvement and conservation efforts, confirming the importance of such diversity for selective breeding programs (Vithya et al., 2021). Moreover, the use of DUS descriptors has been effectively demonstrated in related crops like Indian ginger, strengthening their practical application in enhancing crop productivity and varietal distinctness (Vaishnavi et al., 2024).

Descriptive statistical analysis of rhizome yield traits and biochemical constituents revealed substantial variation among germplasm, consistent with findings reported by previous researchers on *Curcuma* species (Dudekula et al., 2022; Aarthi et al., 2018). Quantitative analyses conducted at the environmentally divergent locations of Coimbatore and Bhavanisagar revealed pronounced genotype-by-environment interactions, providing valuable insights into genotype adaptability and performance. Among the evaluated genotypes, GMN-10 exhibited remarkable consistency across both locations, recording maximum plant height at Coimbatore (69.09 cm) and highest rhizome weight at Bhavanisagar (202.13 g/plant), while maintaining superior essential oil content (0.40% at Coimbatore and 0.50% at Bhavanisagar). Similarly, GMR-2 demonstrated exceptional performance with highest rhizome weight at Bhavanisagar (135.8%) over Coimbatore (88.87 g), positioning it among the top performers. Genotypes cultivated at Bhavanisagar consistently exhibited superior performance for vegetative growth parameters, rhizome dimensions, and yield components compared to those grown in Coimbatore, suggesting that agro-ecological factors exert substantial influence on phenotypic expression and secondary metabolite biosynthesis. These location-specific performance differences can be attributed to variations in edaphic properties including soil fertility, texture, drainage characteristics, and pH balance, as well as climatic parameters such as temperature regimes, humidity patterns, and solar radiation intensity, all recognized as determining factors for optimum black turmeric growth and secondary metabolite synthesis. Genotype GMN-10 showed remarkable stability across locations, performing well for both yield and essential oil content, highlighting its potential as an elite cultivar. These environment-driven variations are supported by findings from Narendhiran et al. (2023), who reported location-specific advantages among black turmeric genotypes based on agro-ecological suitability. Similarly, Dudekula et al. (2022) emphasized the critical influence of genetic and environmental factors on rhizome yield and curcuminoid accumulation in turmeric germplasm. Variation in essential oil content ranging from 0.20% to 0.40% in Coimbatore to 0.40%–0.50% in Bhavanisagar. The high phenotypic coefficient of variation and genotypic coefficient of variation observed for traits including plant height, rhizome dimensions, and essential oil yield indicate substantial inherent variability amenable to effective selection. The close correspondence between phenotypic and genotypic coefficients of variation for most characters suggests predominance of genetic factors over environmental influences in trait expression, a pattern indicative of low genotype-environment interaction and favorable conditions for breeding progress. High heritability estimates for plant height (H^2^ = 99.34% at Coimbatore, 96.99% at Bhavanisagar), leaf morphology, and rhizome yield across both environments imply strong additive genetic effects, ensuring reliability of phenotypic selection in future breeding initiatives. Comparable patterns have been documented in *Curcuma longa* germplasm, where high heritability for rhizome yield and curcuminoid content was associated with stable gene expression across diverse environments (Dudekula et al., 2022).

The GC-MS/MS profiling revealed an extensive array of bioactive metabolites dominated by monoterpenes and sesquiterpenes, with camphor emerging as the principal constituent among the 30 identified phytochemicals. Black turmeric serves as an invaluable source of bioactive phytochemicals, offering diverse therapeutic metabolites with gastroprotective properties including anti-inflammatory, antioxidant, antimicrobial, cytoprotective, and ulcer-healing activities (Chattopadhyay et al., 2018). Natural products demonstrate multi-target mechanisms of action, addressing various pathophysiological aspects of ulcer formation simultaneously, including acid suppression, mucus enhancement, antioxidant protection, and anti-Helicobacter pylori activity (Kumar et al., 2020). The observed genotypic variation in camphor concentration reflects differential regulation of terpene synthase pathways, a phenomenon frequently influenced by complex gene-environment interactions governing secondary metabolism (Bohra et al., 2021). Genotypes including GTE-18 (33.84%), GMR-2 (30.35%), and GMN-10 (26.41%) demonstrated pronounced camphor accumulation, likely attributable to enhanced expression of genes involved in monoterpenoid biosynthesis or favorable allelic variants of rate-limiting enzymes in the mevalonate and methylerythritol phosphate pathways. These findings align with reports demonstrating similar chemotypic differentiation among *Curcuma* accessions exhibiting elevated concentrations of camphor and related sesquiterpenes such as curzerene and epicurzerenone (Benya et al., 2023). The camphor content observed in the present study from sample collected from Odisha (14.60%) differed considerably from previous reports. Ibrahim et al. (2023) documented camphor concentrations ranging from 4.33% to 6.72% in genotypes also collected from Odisha, substantially lower than our findings. Similarly, compositional variations have been reported across different geographical regions. Pandey and Chowdhury, 2003 identified camphor (28.3%) and *ar*-turmerone as the predominant constituents in *C. caesia* rhizome oil, while Mukunthan et al. (2014) reported tropolone (15.86%) as the major metabolites in samples from Southern India. These variations in essential oil composition may be attributed to several factors. Overcultivation and genetic migration of this vegetatively propagated species could contribute to the observed differences in phytochemical profiles. Additionally, terpene metabolites are structurally sensitive and can undergo modifications in response to different environmental conditions. Similar variations in essential oil composition across different geographical locations have been reported by several researchers (Mannu et al., 2020; Ray et al., 2018) highlighting the influence of geographical origin and environmental factors on the essential oil composition of medicinal plants.

The variation in camphor accumulation across cultivation locations reinforces the role of environmental modulation in secondary metabolite biosynthesis. Bhavanisagar conditions favored enhanced essential oil (0.50%) and camphor yield (33.84%), consistent with findings demonstrating that light intensity, temperature regimes, and soil moisture availability significantly influence monoterpene biosynthesis in *C. caesia* (Chitra et al., 2020). This environmental responsiveness indicates phenotypic plasticity, enabling certain genotypes to optimize secondary metabolite production under favorable agro-ecological conditions. Such genotype-environment synergy represents a critical consideration for optimizing both agricultural productivity and industrial phytochemical yield, with implications for site-specific cultivation recommendations and targeted germplasm deployment strategies. The phytochemical composition of *C. caesia*, particularly the presence of flavonoids, tannins, and saponins, has been extensively documented for gastroprotective properties. Flavonoids and tannins function as cytoprotective agents for which antiulcerogenic efficacy has been extensively confirmed through multiple mechanisms. Tannins may prevent ulcer development through protein precipitating and vasoconstriction effects, with their astringent action facilitating microprotein precipitation at ulcer sites, thereby forming an impervious protective layer that hinders gastric secretions and shields underlying mucosa from toxins and irritants. Alkaloids have been shown to prevent stress-induced ulceration (Suresh Arumugam et al., 2011), while ethanolic extracts of *C. caesia* demonstrated significant anti-ulcer potential in aspirin-induced gastric ulcer models, effectively reducing ulcer index, gastric acidity, and pepsin activity while enhancing mucus secretion through prostaglandin-mediated mechanisms (Das et al., 2012).

The molecular docking and dynamics simulations provided mechanistic validation for the anti-ulcer potential of camphor and associated bioactive metabolites through computational analysis targeting key gastric ulcer-associated protein targets including MMP9 (PDB ID: 1ITV), IGFR (PDB ID: 5FXQ), and EGFR (PDB ID: 1WT5), all known for their involvement in gastric mucosal damage and healing mechanisms. MMP9, a crucial enzyme in gastric ulcer pathophysiology, plays a pivotal role in tissue healing and inflammatory response regulation (Dvornyk et al., 2021). Ethanolic extract of *C. caesia* (EECC) confirms its significant anti-ulcer potential in an aspirin-induced gastric ulcer model. The extract effectively reduced ulcer index, gastric acidity, pepsin activity, and enhanced mucus secretion, likely through prostaglandin-mediated mechanisms. These protective effects may be attributed to its rich phytochemical profile, including flavonoids, tannins, and saponins. Thus, *C. caesia* predicted promising gastroprotective activity and supports its traditional use in ulcer management (Das et al.,2012). Comparative evaluation shows that *C. caesia* exhibits anti-ulcer efficacy over the standard drug ranitidine. While ranitidine primarily functions by inhibiting histamine H_2_ receptors to reduce acid secretion, *C. caesia* demonstrated a broader spectrum of gastroprotective actions (Dahi and Almandili, 2021). The *C. caesia* extract significantly reduced gastric volume, total acidity, and pepsin activity, while also enhancing mucus production, a vital defence against mucosal damage. Human ulcer has been shown to be associated with excess acid secretion due to upregulation of the pepsin enzyme. Proteolytic activity of pepsin as the primary aggressor in gastric mucosal ulceration has been reported (Panneerselvam and Arumugam, 2011).

Camphor demonstrated the strongest binding affinity among all tested metabolites, exhibiting a docking score of −7.8 kcal/mol with MMP9, substantially surpassing the standard anti-ulcer reference drug ranitidine (−6.3 kcal/mol). The interaction between camphor and MMP9 involved two hydrogen bonds with active site residues TYR A:111 and TYR A:458, along with stabilizing hydrophobic interactions with TRP A:455 and CYS A:484. Such non-covalent interactions suggest that camphor can modulate MMP9 activity by occupying its catalytic cleft, potentially attenuating matrix degradation and facilitating mucosal repair through preservation of extracellular matrix structural integrity—a mechanism supported by investigations demonstrating MMP9 involvement in ulcer pathogenesis, excessive epithelial degradation, delayed healing, and inflammatory damage (Dvornyk et al., 2021).

EGFR (PDB ID: 1WT5), another critical receptor in gastric healing, was also investigated in this study. EGFR activation promotes epithelial cell proliferation and migration, with EGFR overexpression significantly accelerating gastric ulcer repair through re-epithelialization and oxidative stress reduction *via* MAPK and PI3K-Akt signaling pathways during mucosal regeneration (Shanmugapriya et al., 2021). Camphor exhibited strong binding affinity of −6.8 kcal/mol with EGFR, characterized by stable hydrogen bonding and hydrophobic interactions. Similarly, favorable interactions with insulin-like growth factor receptor suggest that camphor contributes to epithelial regeneration and antioxidative defense mechanisms, consistent with proposed pathways of natural modulators in gastric repair processes (Tarnawski et al., 2014). In contrast, ranitidine interacted with only one residue (GLU A:14) and exhibited lower binding affinity, while camphor demonstrated stronger and more specific interactions with multiple active-site residues. Comparative evaluation revealed that while ranitidine primarily functions through single-mechanism histamine H_2_ receptor inhibition to reduce acid secretion, *C. caesia* bioactives demonstrated a broader spectrum of gastroprotective actions, including reduction of gastric volume, total acidity, and pepsin activity—the primary proteolytic aggressor in gastric mucosal ulceration—while simultaneously enhancing mucus production, a vital defense against mucosal damage (Panneerselvam and Arumugam, 2011).

Further study can be carried to explore this unknown metabolite in subsequent work research in *C. caesia*. In addition to the quantitative analysis, following metabolites such as curzerene, epicurzereneone, curcumeneol, etc., could be found as important in protecting gastrointestinal tract.

## 5 Conclusion

The convergence of high heritability for agronomic traits, substantial biochemical variability, and validated molecular interaction data provides a holistic understanding of *C. caesia’s* genetic and pharmacological potential. The genotypes GTE-18, GMR-2, and GMN-10, characterized by superior rhizome weight, rhizome yield and camphor content and stable performance across cultivation locations in Tamil Nadu, emerge as elite candidates for commercial cultivation and farmer adoption. GC-MS profiling identified camphor as the predominant bioactive constituent alongside curzerenone, epicurzerenone, and various terpenoids, reflecting genetic diversity shaped by regional adaptation. Molecular docking studies revealed that camphor and related phytochemicals exhibited strong binding affinities toward key gastric ulcer-related with binding energies comparable to standard drug. These results complement ethnopharmacological evidence documenting the traditional use of *C. caesia* in treating gastric disorders and provide molecular-level substantiation of its gastroprotective mechanisms through validated computational approaches. However, translation to clinical utility requires comprehensive *in vitro* and *in vivo* validation. This study provides a scientific foundation for advancing *C. caesia* from traditional medicinal systems into evidence-based therapeutic applications for peptic ulcer disease management.

## Data Availability

The original contributions presented in the study are included in the article/Supplementary Material, further inquiries can be directed to the corresponding author.
