# Supplementary material for: Genetic diversity and camphor profiling of Curcuma caesia roxb.: identification of elite genotypes and in silico prediction of gastroprotective mechanisms
Source: Front Pharmacol. 2026 Apr 15;17:1730520. doi: 10.3389/fphar.2026.1730520 (PMC13125016; doi:10.3389/fphar.2026.1730520)
Supplement: Supplementary file 1 [file Table1.docx]

**Supplementary Table 1. Grouping of 21 *Curcuma caesia* genotypes as per the DUS guidelines.**

| **S. No** | **Characterstics** | **Type of Assesment** | **State** | **Score** | **No of genotypes** | **Reference genotypes** |
| --- | --- | --- | --- | --- | --- | --- |
|  | Pseudo stem habit | VG | Compact | 1 | 17 | GAT-1, GMR-2, GKI-3, GRU-4, GCA-5, GKK-6, GAP-8, GCZ-9, GMN-10, GKM-12, GKY-14, GAR-15, GAN-16, GKK-17, GTE-18, GOB-19, GAS-20 |
|  |  |  | Open | 9 | 4 | GMS-7, GND-11, GTM-13, GMU-21 |
|  | Plant height (cm) | MS | Short (<85) | 3 | 18 | GAT-1, GMR-2, GKI-3, GRU-4, GCA-5, GMS-7, GAP-8, GCZ-9, GND-11, GKM-12, GTM-13, GAR-15, GAN-16, GKK-17, GTE-18, GOB-19, GAS-20, GMU-21 |
|  |  |  | Medium (>85-100) | 5 | 1 | GKK-6 |
|  |  |  | Tall (>100) | 7 | 2 | GMN-10, GKY-14 |
|  | Number of leaves | MG | Few (<5) | 3 | 5 | GTM-13, GAN-16, GTE-18, GOB-19, GAS-20 |
|  |  |  | Intermidiate (5-10) | 5 | 12 | GAT-1, GMR-2, GKI-3, GRU-4, GCA-5, GKK-6, GMS-7, GND-11, GKY-14, GAR-15, GKK-17, GMU-21 |
|  |  |  | Many (>10) | 7 | 4 | GAP-8, GCZ-9, GMN-10, GKM-12 |
|  | Leaf lamina length (cm) | MS | Short (<15) | 3 | 0 | - |
|  |  |  | Medium (30-40) | 5 | 9 | GMR-2, GKI-3, GRU-4, GCA-5, GKK-6, GND-11, GTM-13, GAR-15, GTE-18 |
|  |  |  | Long (>40) | 7 | 12 | GAT-1, GMS-7, GAP-8, GCZ-9, GMN-10, GKM-12, GKY-14, GAN-16, GKK-17, GOB-19, GAS-20, GMU-21 |
|  | Leaf lamina width (cm) | MS | Narrow (<10) | 3 | 5 | GMR-2, GCA-5, GCZ-9, GKY-14, GOB-19 |
|  |  |  | Medium (10-15) | 5 | 10 | GAT-1, GKI-3, GRU-4, GKK-6, GMS-7, GND-11, GAN-16, GKK-17, GAS-20, GMU-21 |
|  |  |  | Broad (>15) | 7 | 6 | GAP-8, GMN-10, GKM-12, GTM-13, GAR-15, GTE-18, |
|  | Leaf colour (Ventral side) | VG | Green | 5 | 19 | GAT-1, GMR-2, GKI-3, GRU-4, GCA-5, GKK-6, GAP-8, GCZ-9, GMN-10, GND-11, GKM-12, GTM-13, GKY-14, GAR-15, GAN-16, GKK-17, GTE-18, GAS-20, GMU-21 |
|  |  |  | Dark green | 7 | 2 | GMS-7, GOB-19 |
|  | Leaf colour (Dorsal side) | VG | Light green | 3 | 6 | GMR-2, GRU-4, GKK-6, GKM-12, GAN-16, GMU-21 |
|  |  |  | Green | 5 | 12 | GAT-1, GKI-3, GCA-5, GAP-8, GCZ-9, GMN-10, GND-11, GTM-13, GKY-14, GAR-15, GKK-17, GTE-18 |
|  |  |  | Dark green | 7 | 3 | GMS-7, GOB-19, GAS-20 |
|  | Leaf margin | VG | Even | 3 | 21 | GAT-1, GMR-2, GKI-3, GRU-4, GCA-5, GKK-6, GMS-7, GAP-8, GCZ-9, GMN-10, GND-11, GKM-12, GTM-13, GKY-14, GAR-15, GAN-16, GKK-17, GTE-18, GOB-19, GAS-20, GMU-21 |
|  |  |  | Wavy | 5 | 0 | - |
|  | Leaf venation pattern | MS | Close | 3 | 21 | GAT-1, GMR-2, GKI-3, GRU-4, GCA-5, GKK-6, GMS-7, GAP-8, GCZ-9, GMN-10, GND-11, GKM-12, GTM-13, GKY-14, GAR-15, GAN-16, GKK-17, GTE-18, GOB-19, GAS-20, GMU-21 |
|  |  |  | Distant | 5 | 0 | - |
|  | Leaf midrib colour | VG | Violet blue group | 1 | 13 | GAT-1, GKI-3, GCA-5, GKK-6, GMS-7, GAP-8, GND-11, GTM-13, GKY-14, GKK-17, GTE-18, GOB-19, GAS-20 |
|  |  |  | Purple group | 3 | 6 | GMR-2, GCZ-9, GMN-10, GKM-12, GAR-15, GMU-21 |
|  |  |  | Greyed purple group | 5 | 2 | GRU-4, GAN-16 |
|  | Rhizome habit | VG | Compact | 3 | 8 | GMR-2, GRU-4, GKK-6, GND-11, GKM-12, GTM-13, GAN-16, GOB-19 |
|  |  |  | Intermediate | 5 | 9 | GAT-1, GKI-3, GCA-5, GMS-7, GAP-8, GCZ-9, GKY-14, GKK-17, GTE-18, GAS-20 |
|  |  |  | Loose | 7 | 3 | GMN-10, GAR-15, GMU-21 |
|  | Rhizome shape | MS | Straight | 3 | 8 | GCA-5, GKK-6, GMS-7, GKM-12, GTM-13, GKY-14, GAR-15, GAN-16 |
|  |  |  | Curved | 5 | 13 | GAT-1, GMR-2, GKI-3, GRU-4, GAP-8, GCZ-9, GMN-10, GND-11, GKK-17, GTE-18, GOB-19, GAS-20, GMU-21 |
|  | Rhizome: Number of mother rhizome | MG | One | 1 | 0 | - |
|  |  |  | Two – Three | 3 | 0 | - |
|  |  |  | More than Three | 5 | 21 | GAT-1, GMR-2, GKI-3, GRU-4, GCA-5, GKK-6, GMS-7, GAP-8, GCZ-9, GMN-10, GND-11, GKM-12, GTM-13, GKY-14, GAR-15, GAN-16, GKK-17, GTE-18, GOB-19, GAS-20, GMU-21 |
|  | Rhizome: Status of tertiary rhizome | VG | Absent | 1 | 3 | GAT-1, GCZ-9, GKY-14, GOB-19 |
|  |  |  | Present | 9 | 18 | GMR-2, GKI-3, GRU-4, GCA-5, GKK-6, GMS-7, GAP-8, GMN-10, GND-11, GKM-12, GTM-13, GAR-15, GAN-16, GKK-17, GTE-18, GAS-20, GMU-21 |
|  | Rhizome: Inner core colour | VS | Light blue | 1 | 2 | GMN-10, GKY-14 |
|  |  |  | Greenish blue | 3 | 6 | GMR-2, GRU-4, GAR-15, GKK-17, GTE-18, GMU-21 |
|  |  |  | Moderate blue | 5 | 7 | GKI-3, GCA-5, GKK-6, GTM-13, GAN-16, GOB-19, GAS-20 |
|  |  |  | Strong blue | 7 | 6 | GAT-1, GMS-7, GAP-8, GCZ-9, GND-11, GKM-12 |
|  |  |  | Pale green | 9 | 0 | - |
|  | Duration (Number of Days) | VG | Short (<210) | 3 | 0 | - |
|  |  |  | Medium (211-240) | 5 | 0 | - |
|  |  |  | Long (>241) | 7 | 21 | GAT-1, GMR-2, GKI-3, GRU-4, GCA-5, GKK-6, GMS-7, GAP-8, GCZ-9, GMN-10, GND-11, GKM-12, GTM-13, GKY-14, GAR-15, GAN-16, GKK-17, GTE-18, GOB-19, GAS-20, GMU-21 |

****MG:*** *Measurement by a single observation of a group of plants or parts of plants*

****MS:*** *Measurement of a number of individual plants or parts of plants*

****VG:*** *Visual assessment by a single observation of a group of plants or parts of plants*

****VS:*** *Visual assessment by observations of individual plants or parts of plants*

**Supplementary Table 2: Performance of *C. caesia* germplasm based on quantitative traits in location 1 (Coimbatore)**

| **Genotypes** | **PH** | **NL** | **LL** | **LW** | **DM** | **LR** | **GR** | **NRPP** | **WRPP** | **RYPP** | **EO** |
| --- | --- | --- | --- | --- | --- | --- | --- | --- | --- | --- | --- |
| GAT-1 | 42.89 | 4.00 | 20.40 | 11.18 | 239.50 | 5.90 | 8.18 | 2.35 | 77.57 | 0.82 | 0.30 |
| GMR-2 | 28.64 | 5.10 | 21.39 | 9.07 | 239.00 | 6.59 | 8.21 | 2.92 | 88.87 | 0.92 | 0.40 |
| GKI-3 | 51.94 | 4.70 | 19.01 | 12.10 | 238.00 | 6.10 | 7.43 | 3.23 | 73.25 | 0.73 | 0.30 |
| GRU-4 | 46.65 | 5.00 | 18.89 | 10.06 | 243.00 | 5.60 | 7.72 | 3.05 | 71.25 | 0.77 | 0.25 |
| GCA-5 | 33.98 | 4.60 | 20.22 | 11.80 | 243.00 | 5.16 | 7.09 | 3.37 | 82.41 | 0.88 | 0.30 |
| GKK-6 | 47.30 | 5.10 | 20.69 | 10.47 | 238.00 | 5.78 | 8.18 | 3.17 | 74.77 | 0.69 | 0.25 |
| GMS-7 | 32.89 | 4.60 | 20.91 | 10.21 | 238.50 | 5.98 | 7.02 | 3.88 | 76.83 | 0.79 | 0.20 |
| GAP-8 | 38.87 | 5.30 | 21.09 | 10.57 | 240.50 | 5.51 | 7.34 | 3.62 | 79.24 | 0.66 | 0.25 |
| GCZ-9 | 47.53 | 4.80 | 20.25 | 9.68 | 242.50 | 5.12 | 7.68 | 3.92 | 69.78 | 0.72 | 0.30 |
| GMN-10 | 69.09 | 4.80 | 22.64 | 8.84 | 240.00 | 6.49 | 9.01 | 3.48 | 76.45 | 0.83 | 0.40 |
| GND-11 | 54.31 | 4.80 | 20.41 | 11.49 | 244.00 | 5.85 | 8.80 | 4.25 | 76.41 | 0.69 | 0.30 |
| GKM-12 | 53.37 | 4.40 | 18.42 | 10.90 | 240.50 | 5.54 | 7.01 | 3.55 | 79.12 | 0.83 | 0.25 |
| GTM-13 | 40.41 | 4.81 | 22.06 | 10.99 | 243.00 | 5.89 | 5.61 | 3.73 | 68.80 | 0.86 | 0.30 |
| GKY-14 | 47.55 | 5.30 | 19.53 | 12.61 | 242.00 | 6.47 | 7.52 | 3.63 | 79.12 | 0.90 | 0.25 |
| GAR-15 | 27.81 | 4.70 | 16.50 | 10.75 | 242.50 | 5.52 | 6.26 | 3.90 | 71.00 | 0.78 | 0.20 |
| GAN-16 | 51.68 | 4.80 | 20.80 | 9.01 | 237.50 | 6.72 | 6.92 | 3.87 | 78.19 | 0.83 | 0.25 |
| GKK-17 | 38.93 | 4.20 | 18.75 | 9.89 | 240.50 | 5.87 | 6.85 | 4.28 | 69.42 | 0.83 | 0.30 |
| GTE-18 | 41.10 | 4.90 | 18.75 | 9.53 | 240.50 | 6.53 | 7.47 | 4.45 | 83.06 | 0.89 | 0.40 |
| GOB-19 | 51.66 | 4.90 | 17.23 | 10.05 | 240.50 | 5.87 | 7.74 | 4.32 | 75.27 | 0.86 | 0.30 |
| GAS-20 | 53.33 | 4.80 | 21.89 | 9.72 | 240.00 | 4.42 | 7.13 | 3.88 | 77.10 | 0.83 | 0.25 |
| GMU-21 | 33.66 | 5.00 | 18.45 | 9.89 | 242.00 | 5.59 | 6.67 | 2.63 | 69.68 | 0.73 | 0.30 |
| **Mean** | **44.46** | **4.79** | **19.92** | **10.42** | **240.71** | **5.83** | **7.42** | **3.59** | **76.08** | **0.80** | **0.29** |
| **SD** | 10.08 | 0.32 | 1.59 | 1.02 | 1.89 | 0.55 | 0.80 | 0.56 | 5.13 | 0.07 | 0.06 |
| **Variance** | 101.66 | 0.10 | 2.53 | 1.05 | 3.59 | 0.31 | 0.64 | 0.31 | 26.34 | 0.01 | 0.00 |
| **Min** | 27.81 | 4.00 | 16.50 | 8.84 | 237.50 | 4.42 | 5.61 | 2.35 | 68.80 | 0.66 | 0.20 |
| **Max** | 69.09 | 5.30 | 22.64 | 12.61 | 244.00 | 6.72 | 9.01 | 4.45 | 88.87 | 0.92 | 0.40 |
| **Range** | 41.28 | 1.30 | 6.14 | 3.77 | 6.50 | 2.30 | 3.40 | 2.10 | 20.07 | 0.26 | 0.20 |
| **SE(m)** | 2.20 | 0.07 | 0.35 | 0.22 | 0.41 | 0.12 | 0.17 | 0.12 | 1.12 | 0.02 | 0.01 |
| **SE(d)** | 3.11 | 0.10 | 0.49 | 0.32 | 0.58 | 0.17 | 0.25 | 0.17 | 1.58 | 0.02 | 0.02 |
| **CV-Percent** | 22.68 | 6.66 | 7.98 | 9.82 | 0.79 | 9.51 | 10.77 | 15.52 | 6.75 | 9.35 | 19.72 |
| **CD-0.05** | 6.49 | 0.20 | 1.02 | 0.66 | 1.22 | 0.36 | 0.51 | 0.36 | 3.30 | 0.05 | 0.04 |

*CD@5% - Critical difference at 5% significance level, SE(m) – Standard error of the mean, SE(d) – Standard error of different between means, CV – Coefficient of variation, SD- Standard Variation, Min – Mininum, Max - Maximum*

**Supplementary Table 3: Performance of *C. caesia* germplasm based on quantitative traits in location 2 (Bhavanisagar)**

| **Genotypes** | **PH** | **NL** | **LL** | **LW** | **DM** | **LR** | **GR** | **NRPP** | **WRPP** | **RYPP** | **EO** |
| --- | --- | --- | --- | --- | --- | --- | --- | --- | --- | --- | --- |
| GAT-1 | 80.02 | 6.70 | 44.15 | 12.88 | 242.00 | 8.33 | 10.73 | 4.30 | 180.85 | 1.59 | 0.50 |
| GMR-2 | 80.38 | 7.50 | 35.60 | 8.53 | 249.50 | 8.49 | 10.97 | 4.80 | 198.32 | 1.92 | 0.50 |
| GKI-3 | 83.52 | 6.40 | 35.91 | 12.75 | 242.00 | 7.60 | 9.07 | 5.40 | 169.93 | 1.19 | 0.50 |
| GRU-4 | 81.09 | 7.30 | 35.74 | 12.02 | 244.50 | 7.60 | 10.82 | 4.90 | 190.76 | 1.79 | 0.45 |
| GCA-5 | 79.12 | 6.60 | 34.40 | 8.81 | 241.50 | 7.73 | 8.83 | 5.50 | 158.81 | 1.60 | 0.50 |
| GKK-6 | 91.07 | 7.40 | 34.52 | 12.39 | 240.00 | 8.16 | 11.72 | 4.70 | 192.61 | 1.85 | 0.45 |
| GMS-7 | 81.01 | 7.40 | 48.02 | 12.12 | 245.00 | 7.19 | 8.35 | 5.20 | 142.67 | 1.41 | 0.40 |
| GAP-8 | 79.79 | 7.90 | 50.05 | 16.91 | 240.50 | 7.50 | 11.18 | 5.30 | 184.16 | 1.62 | 0.50 |
| GCZ-9 | 78.41 | 7.70 | 48.15 | 8.78 | 242.00 | 7.63 | 9.30 | 5.30 | 161.66 | 1.76 | 0.50 |
| GMN-10 | 104.32 | 7.90 | 46.54 | 17.16 | 245.00 | 9.50 | 11.94 | 5.00 | 202.13 | 1.92 | 0.50 |
| GND-11 | 78.76 | 6.70 | 35.01 | 12.27 | 243.50 | 8.83 | 10.53 | 5.40 | 199.00 | 1.45 | 0.50 |
| GKM-12 | 78.38 | 7.60 | 47.54 | 16.96 | 240.50 | 7.03 | 10.97 | 5.00 | 186.00 | 1.41 | 0.45 |
| GTM-13 | 79.00 | 4.00 | 36.54 | 18.01 | 241.00 | 6.09 | 9.51 | 5.00 | 194.08 | 1.42 | 0.50 |
| GKY-14 | 107.08 | 7.50 | 47.49 | 8.82 | 241.50 | 7.57 | 11.40 | 5.20 | 185.75 | 1.65 | 0.45 |
| GAR-15 | 77.71 | 7.20 | 34.67 | 16.83 | 244.00 | 6.56 | 6.79 | 3.60 | 135.56 | 1.44 | 0.40 |
| GAN-16 | 80.60 | 4.20 | 48.79 | 12.59 | 242.50 | 8.51 | 12.02 | 5.10 | 190.04 | 1.34 | 0.50 |
| GKK-17 | 80.11 | 7.30 | 48.52 | 12.33 | 240.50 | 7.28 | 9.01 | 5.30 | 178.81 | 1.42 | 0.50 |
| GTE-18 | 80.24 | 4.40 | 35.15 | 13.39 | 242.00 | 8.04 | 10.43 | 5.40 | 191.64 | 1.80 | 0.50 |
| GOB-19 | 78.84 | 3.90 | 49.85 | 8.22 | 240.50 | 7.84 | 9.78 | 4.90 | 176.08 | 1.39 | 0.50 |
| GAS-20 | 81.21 | 4.00 | 46.82 | 12.39 | 244.00 | 7.43 | 11.99 | 4.60 | 183.26 | 1.61 | 0.45 |
| GMU-21 | 80.90 | 7.60 | 46.22 | 12.02 | 244.50 | 7.72 | 8.87 | 5.30 | 164.38 | 1.58 | 0.50 |
| **Mean** | **82.93** | **6.53** | **42.37** | **12.68** | **242.69** | **7.74** | **10.20** | **5.01** | **179.36** | **1.58** | **0.48** |
| **SD** | 8.06 | 1.45 | 6.42 | 3.05 | 2.26 | 0.76 | 1.40 | 0.44 | 18.03 | 0.20 | 0.03 |
| **Variance** | 64.97 | 2.11 | 41.18 | 9.32 | 5.09 | 0.57 | 1.96 | 0.2 | 325.04 | 0.04 | 0 |
| **Min** | 77.71 | 3.90 | 34.40 | 8.22 | 240 | 6.09 | 6.79 | 3.60 | 135.56 | 1.19 | 0.44 |
| **Max** | 107.08 | 7.90 | 50.05 | 18.01 | 249.5 | 9.50 | 12.02 | 5.50 | 202.13 | 1.92 | 0.50 |
| **Range** | 29.37 | 4 | 15.65 | 9.79 | 9.5 | 3.41 | 5.23 | 1.90 | 66.57 | 0.73 | 0.1 |
| **SE(m)** | 1.76 | 0.32 | 1.4 | 0.67 | 0.49 | 0.16 | 0.31 | 0.1 | 3.93 | 0.04 | 0.01 |
| **SE(d)** | 2.49 | 0.45 | 1.98 | 0.94 | 0.7 | 0.23 | 0.43 | 0.14 | 5.56 | 0.06 | 0.01 |
| **CV-Percent** | 9.72 | 22.24 | 15.15 | 24.08 | 0.93 | 9.76 | 13.71 | 8.86 | 10.05 | 12.89 | 7.06 |
| **CD-0.05** | 5.19 | 0.94 | 4.13 | 1.96 | 1.45 | 0.49 | 0.90 | 0.29 | 11.61 | 0.13 | 0.02 |

*CD@5% - Critical difference at 5% significance level, SE(m) – Standard error of the mean, SE(d) – Standard error of different between means, CV – Coefficient of variation, SD- Standard Variation, Min – Mininum, Max - Maximum*

**Supplementary Table 4: Compounds detected in *C. caesia* by GC-MS analysis**

| **S. No** | **Compounds** | **CAS ID** | **Molecular Formula** | **Mol. weight** | **R. time** | **GAT-1** | **GMR-2** | **GKI-3** | **GRU-4** | **GCA-5** | **GKK-6** | **GMS-7** | **GAP-8** | **GCZ-9** | **GMN-10** | **GND-11** | **GKM-12** | **GTM-13** | **GKY-14** | **GAR-15** | **GAN-16** | **GKK-17** | **GTE-18** | **GOB-19** | **GAS-20** | **GMU-21** |
| --- | --- | --- | --- | --- | --- | --- | --- | --- | --- | --- | --- | --- | --- | --- | --- | --- | --- | --- | --- | --- | --- | --- | --- | --- | --- | --- |
|  | 2-Nonanone | [13187](https://pubchem.ncbi.nlm.nih.gov/compound/13187) | [C_9_H_18_O](https://pubchem.ncbi.nlm.nih.gov/#query=C9H18O) | 142.24 | 7.825 | - | - | - | - | - | 0.20 | - | - | - | - | - | - | 0.16 |  | 0.09 | 0.14 | - | - | - | - | - |
|  | Hexadecylmethylglycerol | [10936445](https://pubchem.ncbi.nlm.nih.gov/compound/10936445) | [C_20_H_42_O_3_](https://pubchem.ncbi.nlm.nih.gov/#query=C20H42O3) | 330.5 | 7.830 | 0.05 | 0.07 | 0.11 | - | 0.06 | - | - | - | - | 0.04 | - | - | - | - | - | - | 0.06 | - | 0.07 | 0.11 | 0.10 |
|  | 1,2-Epoxynaonane | [551181](https://pubchem.ncbi.nlm.nih.gov/compound/551181) | [C_9_H_18_O](https://pubchem.ncbi.nlm.nih.gov/#query=C9H18O) | 142.24 | 8.147 | - | - | - | - | - |  | - | - | - | - | - | - | - | - | 0.13 | - | 0.10 | - | - | 0.17 | - |
|  | Camphor | [2537](https://pubchem.ncbi.nlm.nih.gov/compound/2537) | [C_10_H_16_O](https://pubchem.ncbi.nlm.nih.gov/#query=C10H16O) | 152.23 | 9.620 | 7.64 | 20.91 | 9.56 | 10.48 | 13.28 | 0.51 | 20.20 | 11.48 | 5.77 | 15.24 | 7.90 | 2.86 | 5.50 | 12.68 | 7.29 | 0.58 | 4.36 | 14.60 | 8.23 | 3.65 | 9.57 |
|  | Isoborneol | 6321405 | [C_10_H_18_O](https://pubchem.ncbi.nlm.nih.gov/#query=C10H18O) | 154.25 | 9.978 | 2.09 | 4.66 | 3.43 | 4.15 | 4.86 | 0.06 | 7.31 | 2.70 | 2.38 | 3.38 | 2.20 | 1.04 | 1.83 | 3.42 | 2.62 | 0.13 | 1.58 | 3.23 | 2.30 | 1.86 | 3.39 |
|  | Terpinen-4-ol | [11230](https://pubchem.ncbi.nlm.nih.gov/compound/11230) | [C_10_H_18_O](https://pubchem.ncbi.nlm.nih.gov/#query=C10H18O) | 154.25 | 10.317 | - | - | 0.37 | - | 0.27 | 0.03 | - | 0.18 | - | - | 0.18 | - | - | - | - | 0.02 | - | 0.23 | - | 0.31 | 0.39 |
|  | Dimethylglycine-TMS | [13620976](https://pubchem.ncbi.nlm.nih.gov/compound/13620976) | [C_7_H_17_NO_2_Si](https://pubchem.ncbi.nlm.nih.gov/#query=C7H17NO2Si) | 175.3 | 10.460 | - | - | 0.02 | - | - | - | - | - | - | - | 0.01 | - | - | - | - | - |  | - | 0.01 | - | - |
|  | 2,7,7-Trimethylbicyclo[2.2.1] hept | [524272](https://pubchem.ncbi.nlm.nih.gov/compound/524272) | [C_10_H_18_O](https://pubchem.ncbi.nlm.nih.gov/#query=C10H18O) | 154.25 | 10.710 | - | - | 1.02 | 0.34 | 0.70 | 0.10 | 2.24 | - | 1.06 | - |  | 0.43 | 0.58 | - | 0.82 | 0.05 | 0.61 | - | - | 0.91 | 1.29 |
|  | Verbenone | [65724](https://pubchem.ncbi.nlm.nih.gov/compound/65724) | [C_10_H_14_O](https://pubchem.ncbi.nlm.nih.gov/#query=C10H14O) | 150.22 | 10.975 | 0.02 | 0.03 | 0.02 | 0.07 | - | - | - | 0.02 | - | 0.02 | 0.02 | - | - | 0.04 | - | - | - | - | - | - | - |
|  | Carveol | [7438](https://pubchem.ncbi.nlm.nih.gov/compound/7438) | [C_10_H_16_O](https://pubchem.ncbi.nlm.nih.gov/#query=C10H16O) | 152.23 | 11.893 | 0.04 | 0.03 | 0.09 | 0.01 | 0.04 | - | 0.18 | 0.02 | 0.13 | - | 0.08 | 0.04 | 0.06 | 0.02 | 0.07 | - | 0.06 | - | 0.04 | 0.09 | 0.12 |
|  | Guaidiol | [57509397](https://pubchem.ncbi.nlm.nih.gov/compound/57509397) | [C_15_H_26_O_2_](https://pubchem.ncbi.nlm.nih.gov/#query=C15H26O2) | 238.37 | 15.733 | 0.41 | 0.10 | 1.66 | 0.38 | 0.15 | 2.14 | - | 0.15 | 0.64 | 0.11 | 1.40 | 0.90 | - | 0.13 | 0.33 | 1.20 | 0.16 | - | 0.11 | 1.53 | 0.63 |
|  | Humulene epoxide I | [5463721](https://pubchem.ncbi.nlm.nih.gov/compound/5463721) | [C_15_H_24_O](https://pubchem.ncbi.nlm.nih.gov/#query=C15H24O) | 220.35 | 17.352 | 0.05 | 0.05 | 0.05 | 0.04 | 0.04 | 0.24 | - | 0.06 | - | 0.05 | 0.06 | 0.11 | 0.10 | 0.06 | - | 0.13 | 0.02 | 0.04 | 0.04 | 0.02 |  |
|  | Curzerene | [12305301](https://pubchem.ncbi.nlm.nih.gov/compound/12305301) | [C_15_H_20_O](https://pubchem.ncbi.nlm.nih.gov/#query=C15H20O) | 216.32 | 18.460 | 8.02 | 6.72 | 6.24 | 3.53 | 5.26 | 13.12 | 6.05 | 9.27 | 7.25 | 6.87 | 7.50 | 8.43 | 7.94 | 7.83 | 4.90 | 8.70 | 5.24 | 6.78 | 6.51 | 6.40 | 7.56 |
|  | Caryophyllene oxide | [1742210](https://pubchem.ncbi.nlm.nih.gov/compound/1742210) | [C_15_H_24_O](https://pubchem.ncbi.nlm.nih.gov/#query=C15H24O) | 220.35 | 18.589 | - | - | 0.26 | - | 0.04 | - | 0.06 | - | - | - | - | 0.05 | 0.12 | 0.04 | 0.07 | - | 0.06 | 0.11 | 0.04 | 0.02 | 0.06 |
|  | Ageratriol | [181557](https://pubchem.ncbi.nlm.nih.gov/compound/181557) | [C_15_H_24_O_3_](https://pubchem.ncbi.nlm.nih.gov/#query=C15H24O3) | 252.35 | 19.305 | 2.89 | 0.39 | 0.20 | 0.08 | 0.22 | 0.53 | 0.42 | 1.21 | 0.21 | 1.01 | 0.23 | 2.20 | 0.10 | 1.13 | 0.10 | - | 0.05 | 1.04 | 0.35 | - | 0.20 |
|  | Methyl gaMma-linolenate | [6439889](https://pubchem.ncbi.nlm.nih.gov/compound/6439889) | [C_19_H_32_O_2_](https://pubchem.ncbi.nlm.nih.gov/#query=C19H32O2) | 292.5 | 20.450 | - | 0.31 | - | - | - | - | - | - | - | - | - | - | - | - | - | 0.05 | - | - | - | - | - |
|  | Epicurzerenone | [5317062](https://pubchem.ncbi.nlm.nih.gov/compound/5317062) | [C_15_H_18_O_2_](https://pubchem.ncbi.nlm.nih.gov/#query=C15H18O2) | 230.3 | 21.575 | 23.08 | 8.42 | 30.68 | 41.38 | 43.10 | 56.44 | 24.54 | 8.44 | 38.50 | 7.17 | 7.78 | 56.27 | 54.13 | 14.33 | 36.81 | 29.53 | 39.96 | 9.07 | 19.08 | 30.53 | 31.85 |
|  | 5-Formyl-5,8a-dimethyl-2 | [14888882](https://pubchem.ncbi.nlm.nih.gov/compound/14888882) | [C_22_H_34_O_3_](https://pubchem.ncbi.nlm.nih.gov/#query=C22H34O3) | 346.5 | 21.645 | - | - | 1.95 | - | - | - | - | - | - | - | - | - | - | - | - | - | 2.61 | - | 3.08 | 4.01 | - |
|  | Benzoic acid | 243 | [C_7_H_6_O_2_](https://pubchem.ncbi.nlm.nih.gov/#query=C7H6O2) | 122.12 | 21.754 | - | - | 1.08 | - | - | - | - | 0.51 | - | - | - | - | - | - | - | - | - | - | - | - | - |
|  | 1-(2-Hydroxypropan-2-yl)-3a-methyl-6 | [51136415](https://pubchem.ncbi.nlm.nih.gov/compound/51136415) | [C_20_H_34_O_3_](https://pubchem.ncbi.nlm.nih.gov/#query=C20H34O3) | 322.5 | 22.260 | - | - | - | - | - | - | - | - | - | - | 2.88 | - | - | - | - | 3.33 | - | - | - | - | - |
|  | Ledol | [6432561](https://pubchem.ncbi.nlm.nih.gov/compound/6432561) | [C_15_H_26_O](https://pubchem.ncbi.nlm.nih.gov/#query=C15H26O) | 222.37 | 22.47 | - | - | 0.62 | 0.12 | 0.89 | 1.60 | 0.07 | - | 0.07 | - | - | 1.20 | 0.42 | - | - | 0.10 | 0.16 | - | 0.06 | 0.47 | 0.05 |
|  | (-)-Isolongifolol, methyl ether | [91700604](https://pubchem.ncbi.nlm.nih.gov/compound/91700604) | [C_16_H_28_O](https://pubchem.ncbi.nlm.nih.gov/#query=C16H28O) | 236.39 | 22.715 | - | - | - | - | - | - | - | - | - | - | - | - | - | - | 0.22 | 1.44 | - | - | - | 0.39 | - |
|  | 3,7-Cyclodecadien-1-one | [22465744](https://pubchem.ncbi.nlm.nih.gov/compound/22465744) | [C_10_H_14_O](https://pubchem.ncbi.nlm.nih.gov/#query=C10H14O) | 150.22 | 23.188 | 3.59 | 3.46 | - | 25.88 | 9.40 | 12.31 | 3.33 | 3.47 | 3.63 | 4.09 | 4.07 | 7.29 | 6.29 | 7.94 | 2.79 | - | 3.18 | 3.59 | 3.47 | 3.94 | 3.68 |
|  | 14-Hydroxycaryophyllene | [5352484](https://pubchem.ncbi.nlm.nih.gov/compound/5352484) | C[_15_H_24_O](https://pubchem.ncbi.nlm.nih.gov/#query=C15H24O) | 220.35 | 23.413 | - | - | 0.65 | - | 0.28 | - | 0.39 | - | 0.52 | - | - | - | 0.51 | 0.85 | 0.51 | - | - | 1.07 | - | - | 0.60 |
|  | Curcumenol | [167812](https://pubchem.ncbi.nlm.nih.gov/compound/167812) | [C_15_H_22_O_2_](https://pubchem.ncbi.nlm.nih.gov/#query=C15H22O2) | 234.33 | 24.139 | - | - | - | - | - | - | - | - | - | - | 2.98 | - | - | - | 1.08 | - | 2.21 | 0.90 | 0.79 | 1.12 | - |
|  | Longiverbenone | [530428](https://pubchem.ncbi.nlm.nih.gov/compound/530428) | [C_15_H_22_O](https://pubchem.ncbi.nlm.nih.gov/#query=C15H22O) | 218.33 | 25.288 | 36.29 | 40.66 | 20.51 | 4.24 | 7.40 | - | 9.56 | 44.59 | 12.76 | 46.40 | - | 5.09 | 4.96 | 37.01 | 24.52 | - | 25.97 | - | 40.44 | - | 21.13 |
|  | Androstan-17-one, 3-ethyl-3-hydroxy | [14681481](https://pubchem.ncbi.nlm.nih.gov/compound/14681481) | [C_21_H_34_O_2_](https://pubchem.ncbi.nlm.nih.gov/#query=C21H34O2) | 318.5 | 25.957 | - | - | - | - | - | - | 0.11 | - | 2.30 | - | 4.13 | 0.35 | - | - | 2.36 | 1.02 | 0.55 | - | - | 0.99 | - |
|  | Curcumenone | [153845](https://pubchem.ncbi.nlm.nih.gov/compound/153845) | [C_15_H_22_O_2_](https://pubchem.ncbi.nlm.nih.gov/#query=C15H22O2) | 234.33 | 26.088 | - | - | 0.33 | - | 0.14 | - | - | - | - | - | - | 0.18 | 0.17 | 0.23 | - | - | 0.56 | - | - | 0.90 | - |
|  | Zederone | [134687472](https://pubchem.ncbi.nlm.nih.gov/compound/134687472) | [C_15_H_18_O_3_](https://pubchem.ncbi.nlm.nih.gov/#query=C15H18O3) | 246.3 | 29.116 | 0.02 | - | - | - | 0.37 | 2.16 | - | - | - | - | - | - | 0.05 | - | - | 0.42 | - | - | - | - | - |
|  | 7-Hexadecyn-1-ol | [549047](https://pubchem.ncbi.nlm.nih.gov/compound/549047) | [C_16_H_30_O](https://pubchem.ncbi.nlm.nih.gov/#query=C16H30O) | 238.41 | 30.570 | - | 0.11 | - | 0.04 | - | - | - | - | - | 0.08 | - |  | 0.05 | - | - | - | - | - | - | 0.05 | 0.09 |

**ANOVA (Plant height)**

|  | **Df** | **Sum Sq** | **Mean Sq** | **F value** | **Pr(>F)** |
| --- | --- | --- | --- | --- | --- |
| **Genotype** | 20 | 4896 | 244.8 | 0.46 | 0.972 |
| **Replication** | 1 | 0 | 0.0 | 0.00 | 0.996 |
| **Residuals** | 62 | 32995 | 532.2 |  |  |

*(Calculated using R Studio)*
